# Diffusion Tensor Imaging Biomarkers to Predict Motor Outcomes in Stroke: A Narrative Review

**DOI:** 10.3389/fneur.2019.00445

**Published:** 2019-05-08

**Authors:** Luciana M. Moura, Rafael Luccas, Joselisa P. Q. de Paiva, Edson Amaro, Alexander Leemans, Claudia da C. Leite, Maria C. G. Otaduy, Adriana B. Conforto

**Affiliations:** ^1^Neurostimulation Laboratory, Neurology Department, Hospital das Clínicas/São Paulo University, São Paulo, Brazil; ^2^Hospital Israelita Albert Einstein, São Paulo, Brazil; ^3^Lim 44, Department of Radiology and Oncology, Faculdade de Medicina, Hospital das Clínicas/São Paulo University, São Paulo, Brazil; ^4^PROVIDI Lab, Image Sciences Institute, UMC Utrecht, Utrecht, Netherlands

**Keywords:** diffusion MRI (dMRI), diffusion tensor imaging (DTI), corticospinal tract (CST), motor stroke, stroke recovery, white matter (WM)

## Abstract

Stroke is a leading cause of disability worldwide. Motor impairments occur in most of the patients with stroke in the acute phase and contribute substantially to disability. Diffusion tensor imaging (DTI) biomarkers such as fractional anisotropy (FA) measured at an early phase after stroke have emerged as potential predictors of motor recovery. In this narrative review, we: (1) review key concepts of diffusion MRI (dMRI); (2) present an overview of state-of-art methodological aspects of data collection, analysis and reporting; and (3) critically review challenges of DTI in stroke as well as results of studies that investigated the correlation between DTI metrics within the corticospinal tract and motor outcomes at different stages after stroke. We reviewed studies published between January, 2008 and December, 2018, that reported correlations between DTI metrics collected within the first 24 h (hyperacute), 2–7 days (acute), and >7–90 days (early subacute) after stroke. Nineteen studies were included. Our review shows that there is no consensus about gold standards for DTI data collection or processing. We found great methodological differences across studies that evaluated DTI metrics within the corticospinal tract. Despite heterogeneity in stroke lesions and analysis approaches, the majority of studies reported significant correlations between DTI biomarkers and motor impairments. It remains to be determined whether DTI results could enhance the predictive value of motor disability models based on clinical and neurophysiological variables.

## Introduction

Stroke is the second cause of death and the third leading cause of loss of DALYs (Disability-Adjusted Life Years) worldwide. Despite substantial advances in prevention and treatment, the global burden of this condition remains massive (1). In ischemic stroke (IS; 80–85% of the cases), hypoperfusion leads to cell death and tissue loss while in hemorrhagic stroke (HS), primary injury derives from hematoma formation and secondary injury, from a cascade of events resulting in edema and cellular death (2). In IS, cytotoxic edema is a result of glucose and oxygen deprivation, leading to a failure of ion pumps in the cell membranes and consequently to collapse of osmotic regulation, when water shifts from the extracellular to the intracellular compartment (3). In HS, heme degradation products are the primary cytotoxic event and secondarily, an inflammatory process based on degradation of the hematoma takes place (4).

Diffusion MRI (dMRI) is a powerful diagnostic tool in acute IS (5) and is widely used in clinical practice (6). dMRI sequences are sensitive to water displacement. Acute infarcts appear hyperintense on diffusion-weighted imaging (DWI) reflecting the decrease in the apparent diffusion coefficient of water molecules. DWI can be acquired and interpreted over a few minutes. It provides key information for eligibility to reperfusion therapies from 6 to 24 h after onset of symptoms (DAWN study) (7) and in wake-up strokes (8). A search on MEDLINE using the terms “stroke” and “diffusion MRI” yielded 1 article in 1991 and 279, in 2018. Diffusion tensor imaging (DTI) involves more complex post-processing, mathematical modeling of the DW signal (9) and provides measures associated with white matter (WM) microstructural properties (10).

Stroke can directly injure WM tracts and also lead to Wallerian degeneration, the anterograde distal degeneration of injured axons accompanied by demyelination (11). DTI metrics have been studied as biomarkers of recovery or responsiveness to rehabilitation interventions (12–14). The bulk of DTI studies addressed specifically the corticospinal tract (CST), crucial for motor performance or recovery (12, 15), and frequently affected by stroke lesions. Paresis occurs in the majority of the subjects in the acute phase and contributes substantially to disability (16). It is thus understandable that the CST is in the spotlight of research in the field.

Two meta-analyses included from six to eight studies and reported strong correlations between DTI metrics and upper-limb motor recovery in IS and HS (17, 18). In both meta-analyses, heterogeneity between the studies was moderate. In addition, the quality of the evidence of DTI as a predictor of motor recovery was considered only moderate by a systematic review of potential biomarkers (19). The main limitations of the reviewed studies were the lack of cross-validation and evaluation of minimal clinically important differences for motor outcomes as well as the small sample sizes. Heterogeneity in DTI data collection and analysis strategies may also contribute to inconsistencies and hinder comparisons between studies.

In this narrative review, first we review the key concepts of dMRI. Second, we present an overview of state-of-art methodological practices in DTI processing. Third, we critically review challenges of DTI in stroke and results of studies that investigated the correlation between DTI metrics in the CST and motor outcomes at different stages after stroke, according to recommendations of the Stroke Recovery and Rehabilitation Roundtable taskforce (20).

## Concepts of Diffusion MRI

Different MRI paradigms address WM qualitatively and quantitatively (i.e., volume, contrast as signal hyperintensities), but only dMRI allows indirect inferences about WM microstructure by providing information about the underlying organization of the tissue. In regions of little restriction of water displacement (such as the ventricles), water molecules tend to move almost freely (randomly). On the other hand, within tracts, the environment tends to be organized within sets of axons aligned in parallel orientation. Water movement usually follows a specific orientation near axons compactly organized and constrained by the myelin packing (21).

**Figure 1:**
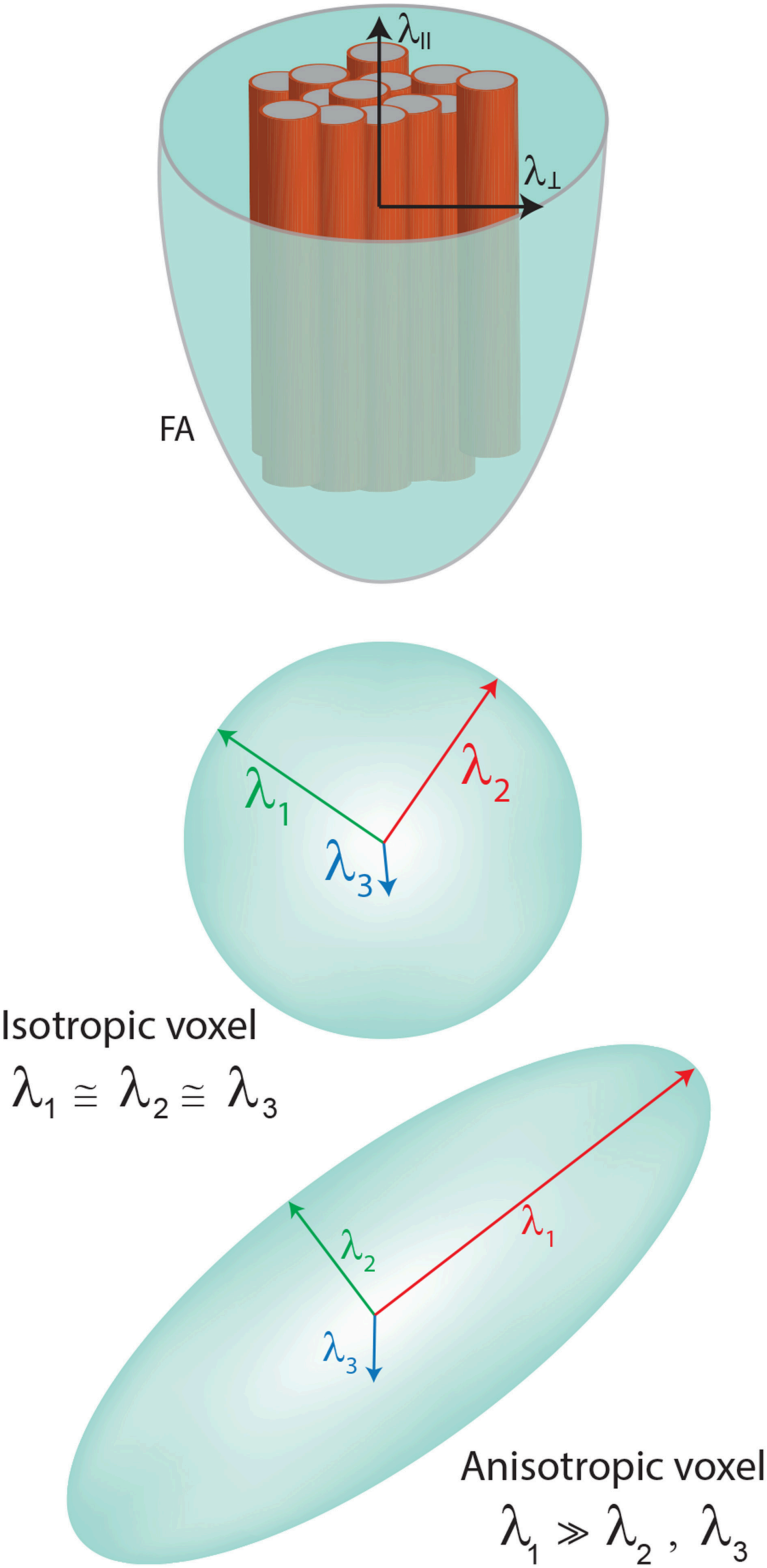
Model of the tensor showing the eingenvalues. Diffusivities are depicted in FA representation (λ_ll_—parallel or axial diffusivity—AD, λ_⊥_perpendicular or radial diffusivity—RD).

The diffusion tensor is the most commonly used mathematical modeling of the diffusion signal and can be decomposed into its eigenvalues (λ) and eigenvectors (ε), required to characterize the signal of water displacement within a voxel. Each eigenvector represents an axis of dominant diffusion with the magnitude of diffusion determined by the corresponding eigenvalues. If the diffusion is isotropic (the same along each orientation), then the eigenvalues have approximately the same magnitude (λ_1_ ≈ λ_2_ ≈ λ_3_), which can be depicted by a sphere. By contrast, if there is a preferential orientation of the diffusion, then the first eigenvalue is bigger than the other two, which can be visualized typically by an ellipsoid (λ_1_ >> λ_2_, λ_3_) (Figure 1).

Hence, the tensor calculation is typically based on a 3 × 3 symmetric matrix, in which the eigenvalues derived from each combination of directions provide different metrics. At least one b0 (non-diffusion-weighted) and 6 non-collinear directions of diffusion-weighted acquisitions are required to minimally describe water displacement with DTI (10). Generally, the more directions, the better.

The most widely used DTI metrics are: fractional anisotropy (FA), mean diffusivity (MD), radial diffusivity (RD), and axial diffusivity (AD). FA describes the degree of anisotropy (represented as an ellipsoid), a value between 0 (isotropic) and 1 (the most anisotropic). Anisotropy tends to increase in the presence of highly oriented fibers (Figure 1). The biggest value is supposed to be found in the center of the tracts. In particular, for CST analysis in stroke or other focal brain lesions, FA results can be reported as ratios between FA extracted from the ipsilesional and the contralesional hemispheres (rFA = FA ipsilesional/FA contralesional). Alternatively, asymmetry in FA can be described (aFA = (FA ipsilesional – FA contralesional)/(FA ipsilesional + FA contralesional).

MD describes the magnitude of diffusion and the biggest value is supposed to be found in the ventricles. RD represents the average diffusivity perpendicular to the first eigenvector and AD is the first eigenvalue (λ1) representing the diffusivity along the dominant diffusion direction.

Many studies have focused exclusively on FA. The proper interpretation of FA often demands knowledge about results of the other three DTI metrics (22). Changes in anisotropy may reflect several biological underpinnings, such as axonal packing density, axonal diameter, myelinization, neurite density, and orientation distribution (21, 23). FA can be decreased in conditions that injure the WM but also when multiple crossing fibers are present in the voxel. In case of partial volume effects, both FA and MD may be altered (24, 25).

### dMRI Acquisition and Processing

DWI is a noise-sensitive and artifact-prone sequence, emphasizing the need for robust acquisitions and processing handling to avoid bias (26). Several dMRI sequences and subsequent post-processing mathematical modeling of the diffusion signal are available. Choices directly impact accuracy, reliability, and validity of the results (27).

dMRI acquisitions and analytical strategies are based on the goal of the study, balancing the pros (i.e., greater reliability of signal reconstruction) and cons (i.e., time-consuming acquisition). In addition to constraints related to the number of subjects with stroke in the studies, criteria to perform a reliable protocol should be weighted prior to data collection [for a review, see Price et al. (28)].

Diffusion images are typically acquired with sequences based on echo planar imaging (EPI) acquisitions. Two high-amplitude magnetic gradients are applied. The *b*-value is a scalar that reflects the degree of diffusion, influenced by the duration, amplitude, and interval between the gradients. *B*-values are comparable to an inverse zoom factor: the higher they are (“high” *b*-values are usually above 1,000 s/mm^2^), the smaller the sampled space (29).

EPI acquisitions are prone to many unexpected distortions (30), therefore care should be taken during data collection. For tensor modeling, some suggestions are: parameters to minimize EPI artifacts; coverage of the entire brain; isotropic voxels; appropriate number of directions and b0s; to acquire at least one low *b*-value (b0 for example), for every 5–6 volumes with high *b*-value and leave it interspersed with those with high values; optimal sampling schemes of the directions in the sphere of distribution and gradient ordering (28, 31). Optimized distribution of gradients can be obtained, for example, with MRtrix software (http://www.mrtrix.org/) or ExploreDTI (http://www.exploredti.com).

Off-resonance artifacts such as eddy currents and magnetic field inhomogeneities are intrinsic to EPI acquisitions and interfere in the expected signal, causing susceptibility-induced distortions (32). Acquisition parameters tailored to prevent and mitigate these artifacts include: parallel imaging; field maps; phase encoding with opposed gradients to correct a geometrical mismatch in the antero-posterior axis; multiple b0s (33). These alternatives demand extra data collection and prolonged scan time (34). In accordance with the chosen acquisition parameters, *a posteriori* corrections are performed in the pre-processing step.

In stroke studies, the duration of scans should be planned by pondering the risk of fatigue and increased head motion in patients with neurologic impairments. These impairments are often not restricted to motor deficits and may involve executive dysfunction or anxiety that contribute to increase head motion and hence, artifacts. Again, trade-offs between “optimal” acquisition parameters, feasibility and noise must be weighted during study design.

Software embedded in the MRI scanner can perform tensor calculations but advanced a posteriori processing is strongly recommended. The most appropriate choice heavily depends on the objectives of the study and on acquisition limitations such as: the number of diffusion directions; image resolution; *b*-values; number of *b*-values; number of averages, repetitions to improve signal in relation to noise and tensor estimation (the number of excitations, NEX) (31, 35).

Many open-source softwares and pipelines are available to process diffusion images, each of them showing particular strengths—a helpful overview can be found in Soares et al. (35). A list of softwares is available on the Neuroimaging Informatics Tools and Resources Clearinghouse (www.nitrc.org). There is no consensus but some agreement about diffusion imaging processing. One can decide to use a mix of softwares to process the data, as long as key steps are completed and a detailed methodological report is made. Documentation is invariably available on-line and discussion forums can provide additional support. It is desirable, to allow reproducibility and comparisons across studies, to transparently report analytical procedures when in-house pipelines are employed (36).

Here, we will briefly cite some suggestions for processing practices, considering an ordinary single-shell acquisition (when only one single *b*-value, in addition to the b0 is acquired) with a *b* value around 1,000 s/mm^2^, with subsequent tensor modeling.

#### Pre-processing

Images must be checked for artifacts, such as susceptibility effects (signal loss and geometric distortions), eddy currents-induced distortions and subject motion (31, 37), so that corrections or exclusions of subjects, volumes or slices are made accordingly. Preferably, automated, quantitative, and not exclusively visual inspection should be performed. Soares et al. (35) provide useful guidelines and a comprehensive list of softwares for quality control.

**Figure 2:**
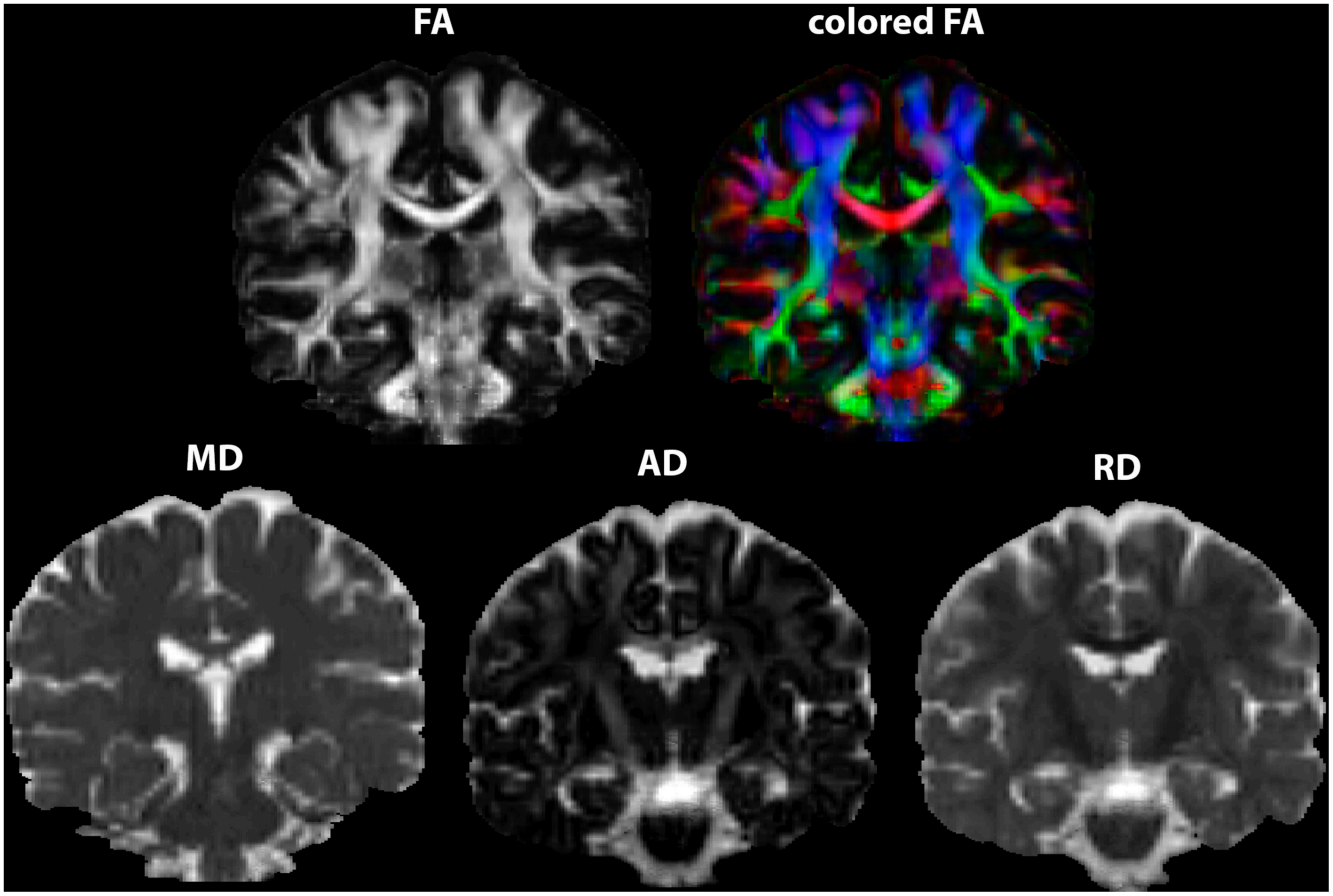
DTI maps generated as output of tensor estimation. FA maps in two versions, the second depicted in RGB colors. Maps were generated in ExploreDTI.

A gold-standard pre-processing pipeline does not exist. Pre-processing is intrinsically dependent on the chosen software. Users can employ different softwares to perform a miscellaneous of corrections, but it is mandatory to follow the basic steps recommended by each developer. Steps of a typical preprocessing pipeline might be:

1. A procedure frequently required, DICOM or PAR/REC conversion to NIfTI format (most diffusion processing softwares use this format).
2. Inspection of DWI images for motion, artifacts (e.g., Gibbs ringing or signal drift) (38, 39) and structural abnormalities: different softwares provide visual and quantitative inspection procedures. It is also important to inspect anatomical images such as T1, T2, and FLAIR.
3. B-matrix rotation: this notion was first introduced by Leemans and Jones (40). The rotation involved in registration of the image must be also applied to the encoding vectors. Neglecting this step may lead to biases in the estimation of the principal vector, affecting all the metrics and tract reconstruction.
4. Brain extraction: an automated segmentation method to delete non-brain tissue from the whole-head. This optional but frequently performed procedure improves registration and normalization.
5. Eddy currents and EPI distortions correction: off-resonance artifacts (as detailed previously) must be corrected. Tools are available, for example, in the ExploreDTI software and in the FSL platform (Topup and Eddy). Further details on how to acquire data and how to perform corrections can be also found at https://fsl.fmrib.ox.ac.uk/fsl/fslwiki/FSL. It must be emphasized that an adequate acquisition is required in order to be able to perform such corrections (41).
6. Tensor estimation in each voxel and generation of maps of FA, MD, RD, and AD (Figure 2). This estimation can be based on different methods and a variety of softwares can perform this calculation but visual inspection of tensor orientation is highly recommended (42–44). If distortions of the expected orientation occur, it is necessary to modify the gradient table, perform reorientation and re-processing, starting over from the first steps (35).

### Post-processing

DTI maps generated in the native space for each subject can be co-registered so that group-wise comparisons can be performed. Co-registration refers to intra or inter-subject spatial alignment of images within or between MRI sequences. Decisions about co-registration tools must consider the paradigm of study, assumptions and specific steps of image processing (45–47). Typical steps of post processing pipeline are highly dependent on the chosen software, but in general, images are co-registered and normalized. Normalization of images to a standard space is a fundamental step to perform comparisons, which is particularly challenging for diffusion images, since they are highly directional and topological (35, 48, 49). After that, group-wise statistics can be performed.

We will review the types of analyses more frequently applied in DTI studies in stroke: ROI-based analysis, tractography, and whole-brain analysis.

### Region-Specific Analysis

#### ROI Analysis

ROIs can be drawn on T1, T2, FA, or ADC images. They can be placed on the abnormal/lesion regions or on predetermined anatomic regions. In the WM, the homogenous signal and EPI distortions might impair robust anatomical delimitation of ROI and reproducibility.

Basic steps of ROI processing are:

1. Registration to improve delineation and to align corresponding voxels in different datasets.
2. Normalization to allow standardized localization and comparisons between subjects within a study. For instance, data from each subject can be transferred to standard space, using a validated template or atlas (such as MNI or Talairach, among others) (34). The choice of the atlas involves checking whether characteristics of the subjects in a given study (i.e., elderly people) are comparable to those of the subjects scanned to build the template (50).
3. Definition of the ROI, manually or semi-automatically. Manual delineation can be achieved by free-hand drawing, by placement of basic shapes such as circles/squares or by drawing of the region. In the former, ROI size differs between subjects while in the latter, it remains constant. Small ROIs may be more specific, but also more prone to errors while large ROIs may be less specific for definition of particular structures and more prone to partial volume effects (inclusion of structures other than the target area) (34).
4. Manual segmentation has high precision but has disadvantages such as the risk of low reproducibility due to dependence on prior knowledge of the researcher and the lack of feasibility of use in large datasets (51). Semi-automated delimitation can be a useful alternative by combining the automated identification of the ROI with a manual, interactive selection and modification by the user (52). Although fully automated delimitation is promising, such as reported by Koyama et al. (53, 54), more studies with large datasets in different phases of stroke are advisable to create a state-of-art automatic method (28, 50–52, 55).
5. Quality control involves: assessment of accuracy of segmentation and registration; report of intra- and inter-rater reliabilities of ROI delineation; clarity of criteria for the location of the ROI (such as anatomical location) - for details, refer to Froeling et al. (34).
6. Extraction of DTI metrics from the ROI, as absolute values from the ipsilesional/contralesional site or ratios between both (56).
7. When more than one ROI is chosen, the correction for multiple comparisons is recommended to reduce false positives—for details, refer to Froeling et al. (34).

#### Tractography

Tractography corresponds to the mathematical reconstruction of tracts (57, 58). By following the preferred direction of water voxel by voxel, it is possible to trace the tracts tree-dimensionally and non-invasively (59, 60). This represents an advantage over ROIs, allowing qualitative and quantitative investigation along of the entire tract of interest. DTI metrics can be extracted from the entire reconstructed tract or from a segment (ROI) of the tract. There are two main approaches for path reconstruction:

1. Deterministic, following the best-fit pathway (the main eigenvector λ1), the principal axis of the tensor aligning with the principal direction of the fibers. It estimates the most likely fiber orientation in each voxel. This method tends to show the best valid/invalid connection trade-offs, but presents low spatial bundle coverage in comparison to the probabilistic method (61).
2. Probabilistic, based on the estimation of uncertainty in fiber orientation (60, 62). It is frequently considered more robust and deals better with partial volume averaging effects, crossing fibers, as well as noise (63). Yet, it is faced with pitfalls, is more time-consuming and computationally expensive.

Noise and artifacts affect reconstructions. There is no “ground-truth” solution to validate tracking results (64). Several efforts are in progress to investigate the ground-truth of diffusion and tracts trajectory by using phantoms, post-mortem, and histological information. The trajectory from the initial (“seed”) voxel to the end point can be represented by a streamline. A streamline refers to the unitary path of reconstruction within a tract and does not indicate an actual nerve fiber or tract (64). Streamlines can vary in different subjects and across experimental paradigms.

Path reconstruction can be constrained by three main steps: seeding, propagation and termination (35). Usually, streamline tractography is based on the placement of multiple ROIs: starting from seed points using a predefined ROI, guiding the path reconstruction by preserving only streamlines passing through or touching other predefined ROIs; full brain tractography keeping the streamlines accordingly with conjunctions, disjunctions, or exclusions ROIs (65). The seeding strategy can also be performed on a voxel-wise level across the brain, running a whole-brain tractography *(e.g., Probtrackx or ExploreDTI)*.

Termination of streamlines is usually guided by a set of parameters: FA threshold (between 0.1 and 0.3 for adult brain), turning angle threshold (depending on the considered tract anatomy—typically between 40 and 70°) to avoid streamlines propagating voxels of high uncertainty, such as the cerebrospinal fluid (CSF) and gray matter (35). Fully automated clustering methods can be alternatives to manual ROI-based approaches (65).

**Figure 3:**
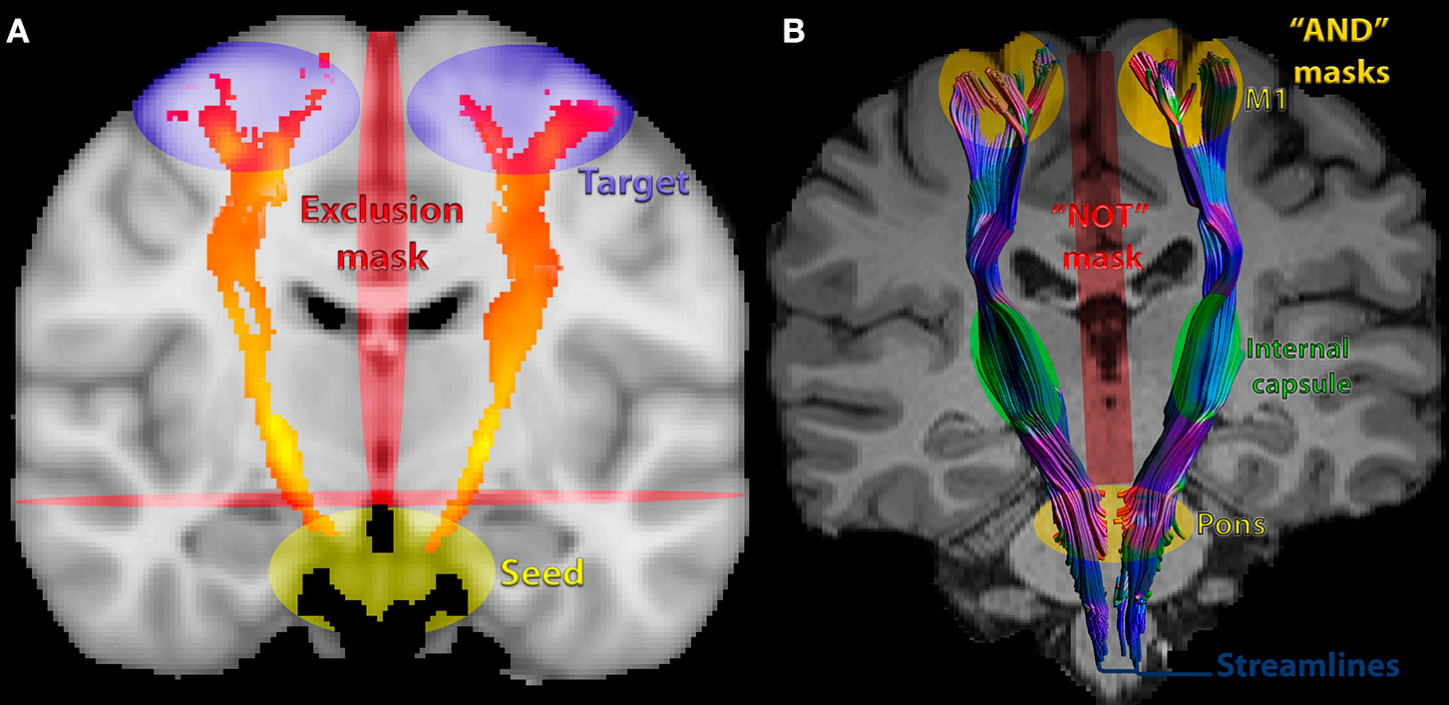
Commonly used seeds, inclusion and exclusions masks for corticospinal tract (CST) DTI-based tractography: **(A)** probabilistic of corticospinal tract (CST) showing commonly chosen masks/ROIs. **(B)** deterministic, showing streamlines. The pons was an inclusion mask in this example. Extraction of metrics can also be performed from this ROI in the pons, in the internal capsule, the entire CST or other parts of the tract.

Several methods of CST reconstruction are available with no consensus. For instance, DTI metrics can be extracted from the entire tract or from ROIs within these tracts, as absolute values from the ipsilesional/contralesional site or as a ratio between both (56). Recently, a DTI challenge of CST reconstruction with tractography demonstrated a consistent presence of false-negative and false-positive pathways. Most of these reconstructions were limited to the medial portion of the motor strip and few were able to trace lateral projections (such as hand-related). Generally, improved results depend on strategies, such as: method of reconstruction, improved signal; sharp estimations of fiber distribution; priors on spatial smoothness; seeding strategies. Anatomically, there are a variety of possible reconstructions, for instance, defined as the pathways coursing through the cerebral peduncles to the pre- and post-central gyrus (61). Park et al. (56) provide detailed information about how to seed and how to confine fibers. Figure 3A shows an example of a probabilistic and Figure 3B, of a deterministic CST tractography.

**Figure 4:**
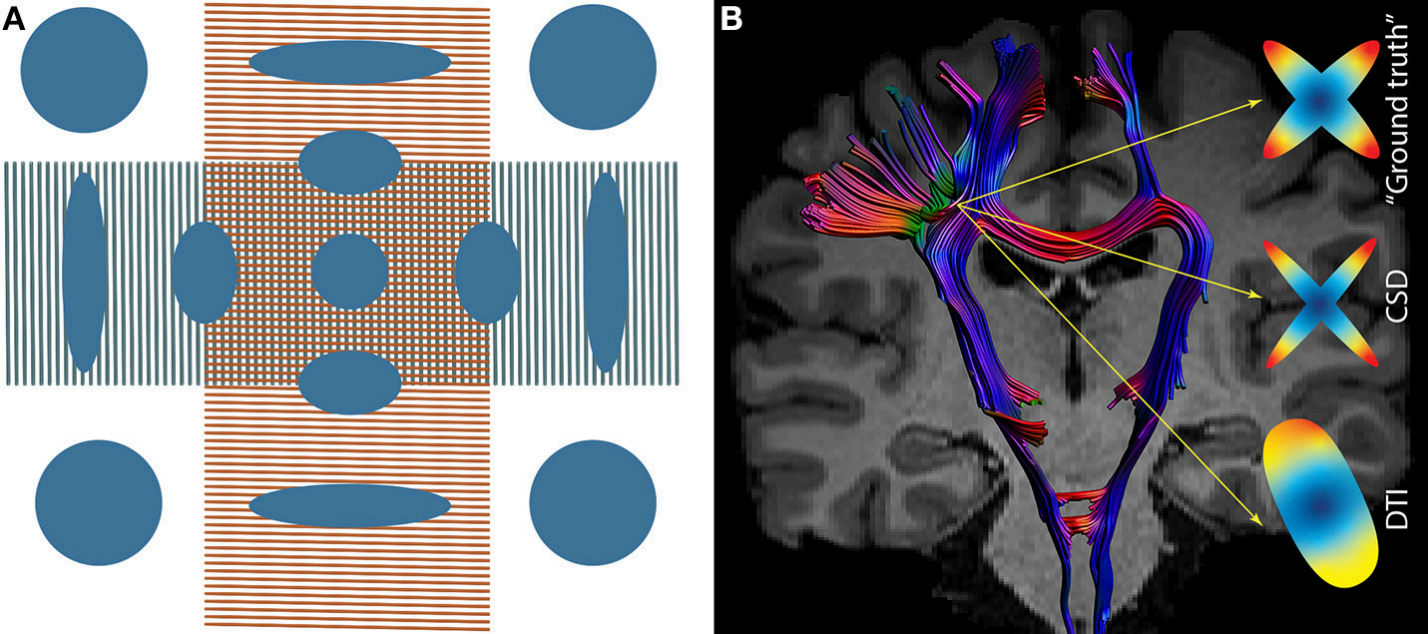
**(A)** Tensor in a region of crossing fibers, when two populations of fibers cross (in this particularly case, at 90 degrees), the tensor fails in the interpretation of the diffusion signal, suggesting low FA (approximately isotropic diffusion). **(B)** Crossing fibers at the centrum semiovale, the ‘ground truth’ signal within a voxel. Constrained Spherical Deconvolution (CSD) depicts two populations of fibers while DTI depicts a single population of fibers.

One of the weaknesses of tensor-based tractography is the assumption that the diffusion related to fibers within a voxel follows a Gaussian distribution, represented by a single direction. This assumption is violated by the presence of crossing fibers and multiple axonal orientations (estimated as ~90% of WM voxels) (66) (Figure 4A). It was hypothesized that increasing the number of directions in the MRI acquisition (such as at least 28 directions in low *b*-values - *b* ~1,000 mm/s^2^) would solve this problem (26, 67). However, it became clear that more advanced models were needed (26, 66).

#### Beyond DTI-Based Tractography: HARDI Models

High angular resolution diffusion imaging (HARDI) uses a larger number of diffusion gradient directions, often in combination with multiple *b*-values, to measure the diffusion signal (68). By doing so, a more reliable reconstruction of the underlying diffusion and fiber orientation distribution can be obtained, overcoming pitfalls such as crossing fibers (Figure 4B). To reach a deeper understanding of the evolution of HARDI models, we refer to Daducci et al. and to Descoteaux et al. (29, 69). HARDI models are superior to DTI to reconstruct the CST (70, 71). However, the higher angular resolution in combination with higher b-values is frequently more time-costly and noisy.

Another approach to model the fiber orientation distribution is Constrained Spherical Deconvolution (CSD) (Figure 4B), typically relying on a single-shell HARDI acquisition and even “low” *b* values in the range of 1,000 s/mm^2^ (72, 73). CSD has medium requirements of acquisition and computation as well as has higher accuracy in fiber orientation estimates than DTI (74). It has been demonstrated that CSD-based tractography consistently reconstructs the fan-shaped CST within the sensorimotor cortex, whereas DTI-based tractography does not (75). Excellent inter-rater and test-retest reliability were reported for FA extracted from CSD-based reconstructions of the CST (76).

#### Whole-Brain Analysis

Whole-brain analysis is an exploratory approach that can be applied to investigate global WM changes or whether such changes are heterogeneous across patients within a study. Analyses can be performed and measures can be extracted using different approaches, such as:

1. Histogram analysis of all voxels in the brain. Histograms that express the frequencies of voxels with a specific value for a DTI metric such as FA can be built. Median, mean, peak height, and peak location of DTI metrics can thus be estimated (59, 77).
2. Brain or WM voxels defined by a mask created from either segmentation of an anatomical MRI or by whole brain tractography. If the former strategy is chosen, DTI values in the voxels can be extracted after registration of anatomical MRI to the non-diffusion weighted image by means of an affine transformation. If whole-brain tractography is performed, then DTI measures can be extracted from voxels that are part of the streamlines.
3. The most popular approach is voxel-based analysis (VBA) and compares DTI metrics in every voxel of the brain (59). This strategy has high reproducibility, is time-efficient and provides excellent spatially localized information, based on the atlases coordinates (78). It provides conservative corrections for multiple comparisons throughout all voxels in the brain, enhancing type II error. Still, it is recommended that corrected results be presented. An alternative is running a cluster-based analysis and correcting them instead of correcting voxel-by-voxel. In addition, novel cluster-based approaches are available to avoid the arbitrary choice of a threshold. TFCE (Threshold-Free Cluster Enhancement) (79) embedded in the tract-based spatial statistics (TBSS - FSL), offers a more robust approach to find significant clusters. TBSS overcomes issues about alignment and smoothing in voxel-based analysis by focusing registration and statistical testing exclusively on the center of the tracts (80). TBSS reduces type II error, at the expense of ignoring findings in the periphery of the tracts. However, TBSS is known to suffer from several methodological limitations that complicate outcome interpretation [for details, see Bach et al. (81)].

## Challenges of DTI in Stroke

### Major Challenge: Heterogeneity of Lesions

The main challenge of DTI in stroke is heterogeneity of lesions—for a review, see de Haan and Karnath (50). Lesion location and size vary across subjects and large lesions often disrupt tracts (80) or promote shifts that impact registration and normalization. In the chronic phase after stroke, loss of brain tissue and secondary dilation of CSF-filled spaces represent an extra-challenge for normalization (82). Special care is advised when inferences are based on large lesions (28). Lesions influence eligibility criteria (so that reliable statistical comparisons between subjects can be made) and impact image processing, demanding a variety of techniques to overcome distortions of the typical anatomy.

The mismatch between images from patients and templates in atlases based on brains from healthy subjects affects normalization (50). Two possible solutions to overcome this mismatch are cost function masking and enantiomorphic normalization. The first approach, which involves masking out voxels of the lesions, may be more useful for small and bilateral lesions. The second approach “replaces” the lesion with brain homolog tissue from the contralesional hemisphere, being useful for large and unilateral lesions placed in symmetric regions, for example as performed by Moulton et al. (83).

Lesion masks can be created by changing the intensity of pixels inside or outside the segmented lesion and hence, obtaining binary images (zero-one intensity). Lesion masks can be manually drawn [for details, see Liew et al. (51)], but several efforts are in course to improve machine-learning algorithms for automatically and accurately segment lesions. Recently, the large open-source T1-weighted dataset ATLAS (Anatomical Tracings of Lesions After Stroke) was released (51). Also, PALS (Pipeline for Analyzing Lesions after Stroke) was developed as a specific tool to improve similarity between manually delimitated lesions. It consists of image reorientation, lesion correction for WM voxels and load calculation, as well as visual inspection of the automated output (84).

Masking out lesions may require large deformations, particularly in WM regions adjacent to gray matter and cerebrospinal fluid. An interesting approach to deal with this problem is DR-TAMAS (Diffeomorphic Registration for Tensor Accurate alignMent of Anatomical Structures) (85) that optimizes normalization by including information not only of FA maps, but of anatomical T1 and T2 images. DR-TAMAS allows creation of atlases based on the diffusion tensor or anatomical images provided by the user. Recently, group-wise registrations without masking lesions were reliably performed on fiber-oriented distribution (FOD)-based algorithms that exclusively rely on diffusion images (CSD-based acquisitions) (83). This approach increased sensitivity to capture FA changes in the CST.

### Challenges for ROI-Based Analysis

The low resolution of DTI images can hinder delineation of the ROI. Registration of the DTI dataset to anatomical T1/T2 images can improve spatial resolution and facilitate ROI drawing. However, misregistration/misalignment can occur, mainly driven by the different distortions in the two types of images and the lower resolution of DTI images resulting in partial volume effects (34, 78). Slight shifts could lead to extraction of metrics from different anatomic regions other than the ROI.

Furthermore, the best choice for ROI placement within the CST remains an open question. According to Koyama et al. (53), outcome prediction is more accurate when fully automated ROIs are placed in the cerebral peduncle. According to Park et al. (56), the extraction of DTI measures from the posterior limb of internal capsule (PLIC) is reliable. Tang et al. (86) reported that ROIs in the brain stem are more subjected to partial volumes problems (caused by the proximity with CSF) than at the PLIC.

### Challenges for Tractography

In stroke, tractography may be used to reconstruct a tract of interest based on a prior hypothesis, to obtain qualitative anatomical information (visual evidence of disruption of the tract), extract quantitative measures (volumetric and diffusion metrics) or make inferences about connectivity (87).

To track the CST, a standard template based on healthy subjects can be reliably used to extract metrics from the whole-tract or from a section of it, such as within the PLIC (56). In strokes that affect the CST, tractography may not be feasible because of the loss of normal pathway of axons within the tract, leading to an unreliable morphology of the tracts (64). In turn, the placement of individual ROIs can be problematic because it is operator-depending biased, time-consuming, limited in feasibility and generalizability. For this reason, the use of a template from healthy volunteers to guide extraction is a possible alternative (56). Limitations regarding anatomical accuracy and quantitative evaluation of tractography in stroke should be considered [for details, please see Jbabdi and Johansen-Berg (88); Thomas et al. (89)].

### Challenges for Whole-Brain Analysis

Typical steps of whole-brain processing pipeline involve co-registration and normalization so that group-wise statistical comparisons can be made. Stroke lesions can be obstacles for automatic whole-brain voxel-wise analysis such as TBSS. The cost function masking and enantiomorphic normalization can be used as alternatives to overcome lesion deformations.

### Challenges for Replicability

Results are dependent on the adoption of good practices regarding acquisition parameters, pre and post-processing. Researchers may tend to use their own tools or manual methods (84), but guidelines to improve repeatability and reproducibility are available, such as those made available by the Quantitative Imaging Biomarkers Alliance (QIBA) http://qibawiki.rsna.org/index.php/Main_Page. Also, it is crucial to use the same package and software version within the same study and while processing longitudinal datasets. Whenever possible, the most updated version should be chosen (64).

## dMRI as a Biomarker of Recovery in Stroke

In this section, we review studies that assessed correlations between DTI measures on the CST to predict motor recovery.

LMM and RL searched MEDLINE (Medical Literature Analysis and Retrieval System Online; through the PubMed interface) and Web of Science, using the following keywords: motor (stroke or infarct or infarction or hemorrhage) and corticospinal tract and diffusion (imaging or tensor imaging). A complementary search was made using the first two keywords combination and tractography or FA. Studies were selected according to the following criteria.

Inclusion criteria: evaluation of patients with IS or HS; publication from January, 2008 until December 5th, 2018; collection of MRI data for DTI metrics in the hyperacute (< 1 day after onset of symptoms) (Table 1), acute (2–7 days) (Table 2), or early subacute (7 days−3 months) (Table 3) phases after stroke, according to definitions of the Stroke Recovery and Rehabilitation Roundtable taskforce (20); original articles; evaluation of at least one DTI metric (FA, AD, RD, or MD) in the CST; prospective assessment of motor outcomes (at least 4 weeks after stroke) with measures of body structure and function (such as the Medical Research Council Scale, NIH Stroke Scale, Motricity Index of Arm and Leg, Fugl-Meyer Motor Assessment, among others) or with measures of activity (such as the Action Research Arm Test, or Wolf Motor Function Test), according to the International Classification of Functioning, Disability and Health (ICF)—WHO 2001—http://www.who.int/classification/icf/en/ (90); evaluation of correlations between DTI metric(s), and motor outcomes (but not changes in motor outcomes relative to baseline); minimal sample size, 10 patients; post-processing of images performed with whole-brain, ROI (region-of-interest) or tractography strategies. Studies that performed tractography but did not report DTI metrics were excluded. Cross-sectional studies were not included in the review.

**Table 1:** Studies that correlated DTI metrics on the CST in the hyperacute phase (first 24 h) and motor outcomes assessed at least 4 weeks after stroke.

**Study**	**Lesion characteristics**			**Demographics**				**Details of acquisition**			**Technique/** **software/** **metrics**	**Mentioned lesion mask**	**CST**		**Time of evaluation (months after stroke)**	**Reported rehabilitation**	**Outcome**	**Did CST DTI metrics correlate with outcomes?**
	**Type**		**Lesion site or arterial territory if IS**	***n***	**Gender M/F**	**Age range**	**Mean age (SD)**	**MRI field (T)**	**Directions/b0**	**b value (s/mm^2^)**			**Ipsilateral**	**Contralateral**				
	**IS**	**HS**																
Ma et al. (91)	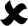	**√**	Basal ganglia	23	15/8	34–67	54 (9)	1.5	6/NS	1,000	ROI: CP ipsilesional Volume-One, dTV FA	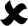	**√**	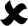	3	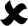	NIHSS_m_	FA: yes *r* = −0.926
Kusano et al. (92)	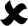	**√**	Thalamus, putamen	18^*^	11/7	30–99	67.8 (16.2)	3.0	6/NS	1,000	ROI: anterior CP SPM99 FA, MD, rFA	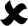	**√**	**√**	1	**√**	PG mRS BI	rFA: yes PG, *r* = −0.767 mRS, *r* = −0.676 BI: no
Puig et al. (93)	**√**	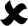	Middle Cerebral Artery	60	38/22	45–85	68 (13)	1.5	15/NS	1,000	ROI: rostral pons DTIWeb 2.0 FA, rFA	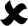	**√**	**√**	1	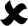	NIHSS_m_	No

*NS, not specified; IS, ischemic stroke; HS, hemorrhagic stroke; CST, corticospinal tract; r, ratio between ipisilesional/contralesional; ROI, region of interest; CP, cerebra peduncle; FA, fractional anisotropy; MD, mean diffusivity; PG, Paresis Grading; NIHSSm, National Institutes of Health Stroke Scale; mRS, Modified Rankin Scale; BI, Barthel Index*.

* *13 subjects were scanned within 0–1 day and 5 within the second day post-stroke*.

**Table 2:** Studies that correlated DTI metrics on the CST in the acute phase (days 2–7 post-stroke) with motor outcomes assessed at least 4 weeks after stroke.

**Study**	**Lesion characteristics**				**Demographics**				**Details of acquisition**			**Technique/** **software/** **metrics**	**Mentioned lesion mask**	**CST**		**Time of evaluation (months after stroke)**	**Reported rehabilitation**	**Outcome**	**Did CST DTI metrics correlate with outcomes?**
	**Type**		**Time period after stroke for DTI acquisition (days)**	**Lesion site or arterial territory if IS**	***n***	**Gender M/F**	**Age range**	**Mean age (SD)**	**MRI field (T)**	**Directions/b0**	**b value (s/mm^2^)**			**Ipsilateral**	**Contralateral**				
	**IS**	**HS**																	
Yoshioka et al. (101)	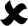	**√**	2–5	Thalamus, putamen	17	12/5	49–74	61.8 (NS)	1.5	13/NS	1,000	Tractography (NS) dTV II and VOLUME ONE ROI: CP to precentral gyrus FA, rFA	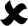	**√**	**√**	3	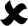	MMT	rFA: yes *r* = 0.55 *p* < 0.05
Puig et al. (93)	**√**	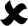	3	Middle cerebral artery	60	38/22	45–85	68.2 (13.6)	1.5	15/NS	1,000	ROI: rostral pons DTIWeb 2.0 FA, rFA	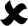	**√**	**√**	1	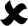	NIHSS_m_	No
Kuzu et al. (102)	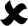	**√**	3	Thalamus, putamen	23	12/11	44–85	65 (13)	3.0	6/1	800	ROI: CP Functoll (GE) FA	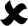	**√**	**√**	3	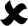	NIHSS_m_ (3 m)	FA: yes (CC not given; *p* = 0.006)
Wang et al. (95)	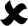	**√**	3	Thalamus, putamen	27	14/13	42–77	60.2 (10.5)	1.5	15/NS	1,000	ROI: CP SPM99 rFA, rMD	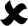	**√**	**√**	6	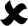	PG (6 m) mRS (17 m) FIM (17 m)	rFA: yes PG, *r* = −0.642 mRS, *r* = −0.549 FIM, *r* = 0.532
Groisser et al. (94)	**√**	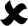	3–7	Middle cerebral artery	10	5/5	19–67	53 (13)	3.0	60/10	700	Tractography (NS) ROI: 10 voxels with highest density Trackvis r (FA, AD, RD)	**√**	**√**	**√**	6	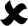	Hand grip MI NHPT	rAD: yes Hand grip, rs = 0.85 MI, rs = 0.97 NHPT: no rFA and rRD: no
Doughty et al. (103)	**√**	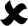	2	Cerebral hemisphere	58	24/34	NS	61.3 (14.2)	1.5	30/1	1,000	ROI: CP and N5S SPM and MRIcro, aFA	**√**	**√**	**√**	3	**√**	FMA	aFA, N5S: yes aFA, CP: no

*NS, not specified; IS, ischemic stroke; HS, hemorrhagic stroke; CST, corticospinal tract; r, ratio between ipisilesional/contralesional; FA, fractional anisotropy; aFA, asymmetry between (contralesional-ipsilesional)/(contralesional+ipsilesional) fractional anisotropy; MD, mean diffusivity; AD, axial diffusivity; RD, radial diffusivity; CC, correlation coefficient; ROI, region of interest; CP, cerebra peduncle; PLIC, Posterior Limb of the internal capsules; N5S, nearest 5 slices; aFA, FA asymmetry; PG, Paresis Grading; MMT, Manual Muscle Test; NIHSSm, National Institutes of Health Stroke Scale; mRS, Modified Rankin Scale; NHPT, Nine Hole Peg Test; FIM, Functional Independence Measures; FMA, Fugl-Meyer Motor Assessment; MMT, Manual Muscle Testing*.

**Table 3:** Studies that correlated DTI metrics within the corticospinal tract (CST) in the early subacute phase (7–90 days post-stroke) with motor outcomes assessed at least 4 weeks after stroke.

**Study**	**Lesion characteristics**				**Demographics**				**Details of acquisition**			**Technique/software/metrics**	**Mentioned lesion mask**	**CST**		**Time of evaluation (months after stroke)**	**Reported rehabilitation**	**Outcome**	**Did CST DTI metrics correlate with outcomes?**
	**Type**		**Time period after stroke for DTI acquisition (days)**	**Lesion site or arterial territory if IS**	***n***	**Gender M/F**	**Age range**	**Mean age (SD)**	**MRI** **field (T)**	**Directions/b0**	**b value (s/mm^2^)**			**Ipsilateral**	**Contralateral**				
	**IS**	**HS**																	
Radlinska et al. (104)	**√**	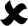	12	Included white matter	18	5/13	42–86	73.0 (12.9)	3.0	64/NS	1,000	Tractography (NS) MINC suite tools rFA	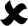	**√**	**√**	6	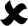	RMFT	rFA: yes *r* = 0.87
Koyama et al. (105)	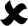	**√**	14–18	Thalamus, putamen or both	15	6/9	31–85	51 (NS)	3.0	12/1	1,000	ROI: CP Customized code IDL software rFA		**√**	**√**	1	**√**	MRC (1 m)	rFA:yes *r*^2^ = 0.272
Wang et al. (95)	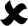	**√**	14	Thalamus, putamen	27	14/13	42–77	60.2 (10.5)	1.5	15/NS	1,000	ROI: CP SPM99 FA, MD	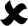	**√**	**√**	6	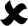	NIHSS_m_ (6 m) mRS (17 m) FIM (17 m)	rFA: yes NIHSS_m_, rs = −0.7 mRS, rs = −0.653 FIM, rs = 0.661
Kim et al. (106)	**√**	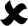	7–30	Middle cerebral artery	37	28/9	27–81	57.4 (15.2)	1.5	32/NS	1,000	Tractography (NS) FSL, DTI-Studio ROI: anterior mid-pons to anterior lower pons rFA	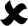	**√**	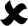	6	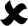	MI MBC	rFA: yes MI, *r* = 0.517 MBC, *r* = 0.473 *p* = 0.003
Koyama et al. (53)	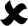	**√**	14–18	Thalamus, putamen	32	16/16	31–88	NS	3.0	12/1	1,000	ROI: CR/IC and CP FSLutils rFA	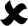	**√**	**√**	1	**√**	MRC mRS (1 m)	rFA: yes CP, MRC, *r*^2^ = 0.271 CP, MRS, *r*^2^ = 0.239 CR/IC,MRC, *r*^2^ = 0.085 CR/IC, MRS, *r*^2^ = 0.057
Puig et al. (12)	**√**	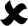	30	Middle cerebral artery	70	42/28	NS	72 (12)	1.5	15/NS	1,000	ROI: pons NeuroScape 2.0 MR Stroke Edition rFA	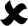	**√**	**√**	24	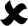	MI (2 y)	rFA: yes (OR = 1.6)
Groisser et al. (94)	**√**	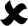	30–60	Middle cerebral artery	10	5/5	19–67	53 (13)	3.0	60/10	700	Tractography (NS) ROI: 10 voxels with highest density Trackvis r (FA, AD, RD)	**√**	**√**	**√**	6	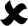	Hand grip MI NHPT (6–7 m)	rFA: yes Hand grip, rs = 0.7 MI, rs = 0.87 NHPT, rs = 0.79 rAD and rRD: No
Koyama et al. (97)	**√**	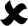	14–18	Cortex ± corona radiata or PLIC	16	11/5	47–80	70 (NS)	3.0	12/1	1,200	Whole-brain voxel-wise analysis TBSS, ROI = CST FSL FSLutils of CST FA, rFA	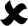	**√**	**√**	~3	**√**	BRS FIM (5–7 m)	rFA and BRS: yes Proximal, rs = 0.687 Distal, rs = 0.579 rFA and FIM: No
Cheng et al. (107)	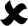	**√**	14	Thalamus, putamen	48	31/17	NS	62 (14)	3.0	30/NS	1,000	Tractography (NS) ROI: CR, CP, pons, PH Neuro 3D software FA, rFA	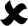	**√**	**√**	3	**√**	MI	rFA at CR after 1 and 3 months: yes MI at 1 m, rs = 0.433 MI at 3 m, rs = 0.405 CP, pons, PH: No
Imura et al. (108)	**√**	**√**	10	NS	25	14/11	NS	71.5 (11)	3.0	16/NS	1,000	Tractography (NS) FiberTrak FA	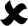	**√**	**√**	1	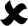	MI BRS	FA, CST: Yes MI-UE, rs = 0.65 BRS-UE, rs = 0.61 MI-LE, rs = 0.60 BRS-LE, rs = 0.69
Zhang et al. (109)	**√**	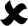	14	Pons	17	12/5	34–73	58.3 (NS)	3.0	64/1	700	4 ROIs (medulla, CP, IC, CS) DT imaging studio rFA	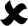	**√**	**√**	6	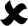	FMA mRS (3 m, 6 m)	rFA, ROIs CP, IC,CS: yes 3 m FM, *r* = 0.771 rFA, mRS, *r* = −0.569 6 m FM, *r* = 0.73 mRS, *r* = −0.498 ROI medulla: No
Buch et al. (110)	**√**	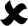	14	Middle and/or anterior cerebral artery	25	14/11	37–83	61 (NS)	3.0	30/1	1,000	Whole-brain voxel-wise analysis TORTOISE, DTI-TK, MRIcro aFA	**√**	**√**	**√**	3	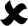	FMA	Yes rs < −0.8 *p* < 0.0001
Jang et al. (100)	**√**	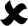	7–28	Pons	31	12/19	36–79	64.76 (10.76)	1.5	32/NS	1,000	Tractography (deterministic) ROI: pons DTI studio FA, rFA	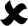	**√**	**√**	6	**√**	MI BRS	FA, rFA: no

*NS, not specified; IS, ischemic stroke; HS, hemorrhagic stroke; M, male; F, female; SD, standard deviation; T, Tesla; CST (corticospinal tract); UE, upper extremity; LE, lower extremity; FA, fractional anisotropy; rFA, ratio between ipisilesional/contralesional fractional anisotropy; aFA, asymmetry between (contralesional-ipsilesional)/(contralesional+ipsilesional) fractional anisotropy; CS, centrum semiovale; ROI, region of interest; CR, corona radiata; IC, internal capsule; CP, cerebra peduncle; TBSS, tract-based spatial statistics; PH, parihoematomal edema; MBC, Modified Brunnstrom classification; BRS, Brunnstrom Recovery Scale; MRC, Medical Research Council; r, Pearson correlation coefficient; rs, Spearman correlation coefficient; OR, odds ratio; NIHSSm, Motor score (upper and lower limb); mRS, Modified Rankin Scale; NHPT, Nine Hole Peg Test; MI, Motricity Index; FIM, Functional Independence Measures; FMA, Fugl-Meyer Motor Assessment*.

The following information was retrieved from the manuscripts (Tables 1–3): type of stroke; lesion site or affected arterial territory; number of subjects; age; gender; MRI field; number of directions/b0; *b* value (s/mm^2^); methods of analysis (technique/software/metrics); whether lesion masks were mentioned; whether ipsilesional and contralesional CST were assessed; when motor evaluation was performed (time from stroke); motor outcome; whether DTI correlated with outcome and correlation coefficients.

A total of 425 manuscripts were retrieved and 354 were excluded based on the title or abstract (Supplementary File and Supplementary Figure); 71 manuscripts were read, 52 were excluded and 19, included in the review. The results are summarized according to the phase after stroke in which MRIs were performed: hyperacute (<12 h, Table 1), acute (2–7 days, Table 2), and early subacute (>7–90 days, Table 3). One study (Puig et al., *n* = 60) (93) performed measures in the hyperacute and acute, and two—Groisser et al., *n* = 10 (94); Wang et al., *n* = 27 (95) in the acute and subacute phases. A total of 570 subjects were included in the selected studies: three (*n* = 101) in hyperacute, six (*n* = 172) in the acute and nine (*n* = 297), in the early subacute phase after stroke. A total of 667 scans were performed. Ages ranged from 19 to 99 years and 56.5% of the patients were men. 62.8% of the patients had IS and 37.2%, HS. 36.8% of the studies reported that patients received rehabilitation during the time between the MRI scan and the evaluation of motor outcomes. All of the studies reported at least one metric of body structure and function and 47.4%, at least one metric of activity according to the ICF. The Motricity Index, an ordinal measure of limb strength (1–100) (96), was the most widely used scales for assessment of motor performance.

MRI scans were performed on 3T scanners in 57.9% of the studies. The number of directions during diffusion acquisitions ranged from 6 to 64 and the number of b0, from 1 to 10. 83.3% of the studies used b values of 1,000 s/mm^2^.

Only 15.8% of the studies explicitly mentioned lesion masks during pre-processing and 18 different softwares were used for data analysis. 52.6% measured DTI metrics according to ROI-based methods, 36.8%, according to ROI in tractographies, and 10.5% within the entire CST according to tractography; 10.5% extracted the entire CST as a ROI based in whole-brain processing in TBSS (97). The most commonly chosen ROIs were the cerebral peduncle (61%) and the pons (33%).

Despite great heterogeneity in methods of collecting and analyzing the data, the majority of studies reported statistically significant correlations between DTI biomarkers and motor outcomes: 66.7% in the hyperacute, 83.3% in the acute, and 92.3% in the early subacute phases after stroke. Motor impairments were evaluated from 4 weeks to up to 6 months later in the hyperacute/acute studies, and up to 2 years in the subacute studies. DTI results closer to normal, from the 1 day up to 3 months after stroke, were correlated with less severe impairments.

FA, rFA, or aFA were measured in 100% of the studies. At least one of these metrics was significantly correlated with motor outcomes in 66.7% of hyperacute or acute, and in 92.3% of early subacute studies. FA values vary across subjects and are influenced not only by the stroke, but also by subclinical white matter lesions that are frequent in patients with vascular disease in the ipsilesional as well as in the contralesional hemisphere (98, 99). However, the changes in FA values in the CST due to chronic white matter lesions is expected to be less severe than those caused by stroke. None of the identified studies reported discrepant results in regard to correlations between clinical outcomes and FA metrics (for instance, correlation of outcomes with rFA but not with FA). Two studies (93, 100) reported absences of correlations between clinical outcomes and FA or rFA. Other studies that described correlations between rFA or aFA and motor outcomes did not mention whether correlations were also present between ipsilesional FA and outcomes (Tables 1–3). Therefore, it is not possible to define whether measures of asymmetry are more strongly correlated to motor outcomes, when compared to absolute ipsilesional FA values.

Puig et al., Groisser et al., and Jang et al. did not find significant correlations between FA metrics and motor outcomes at some of the stages (93, 94, 100).

Puig et al. (93) assessed FA and did not find a significant correlation between this measure < 12 h or at 3 days, or impairments at 3 months, in 60 patients after stroke. In this study, there was no significant asymmetry in FA values for the CST (ROI: pons) measured hyperacutely or at 3 days post-stroke, but there was a significant asymmetry 1 month later. FA abnormalities at 1 month correlated with motor performance also assessed at 1 month. Only MCA infarcts were included, and it is possible that measurements extracted from the CST at the pons, away from the infarcts at a time when Wallerian degeneration might not yet fully ensued, may have contributed to this negative finding (93).

Groisser et al. measured not only FA, but also AD, MD, and RD in 10 patients at 3–7 days and 1–2 months post-stroke. Only asymmetries in AD at 3–7 days correlated with hand grip and the Motricity Index at 1–2 months, but not with performance in the nine-hole peg test. In contrast with other studies, the authors performed tractography and evaluated DTI measurements in the 10 voxels with the highest CST density ipsilesionally. This methodological difference may have contributed to the discrepant result, particularly in subjects with large lesions: 70% of the patients had large infarcts (>1/3 middle cerebral artery territory) (94). DTI analysis can be challenging in subjects with large lesions, especially in the acute phase when edema and MD reduction are at a peak (3). Only another study included patients with MCA infarcts at this stage (103): 58 subjects were tested, with a mean infarct volume of 39 ml, and a significant correlation was found between FA asymmetry measured at 2 days and the Fugl-Meyer Motor Assessment measured at 3 months, when measurements were made at the nearest-5-slices ROI, but not at the cerebral peduncle.

On the other hand, Groisser et al. found that changes in FA measured at a later stage (1–2 months) correlated with hand grip, Motricity Index and nine-hole peg test measured at 6 months, in line with other studies that assessed DTI at the early subacute phase post-stroke (94).

Jang et al. were the only authors who did not report correlations between FA or rFA at the early subacute phase, and motor impairments. Only subjects with pontine infarcts were included, and measures were made at the pons, from 7 to 28 days post-stroke, according to tractography. The authors hypothesized that lack of a significant difference in the directionality of the residual CST at this level may have contributed to this finding (100).

Few of the selected studies measured AD, MD, and RD (83, 111, 112). FA is a highly sensitive, but quite non-specific measure (22, 113). Nevertheless, the results of this narrative review suggest a consistent relation between FA measured in the CST at early stages after stroke, and motor impairments, in line with results of meta-analyses (17–19). However, studies included in this review predominantly assessed motor impairments, rather than activity (disability) according to the ICF. It remains to be clarified if DTI measures within the first hours to 3 months after stroke can predict long-term disability.

A key question is whether DTI results enhance the predictive value of models of motor disability based on clinical information such as age and motor impairments, and neurophysiological testing. For instance, Stinear et al. reviewed data from 207 patients clinically assessed for upper limb impairments (SAFE score: shoulder abduction and wrist extension) and overall neurological impairments (NIH stroke scale) within 3 days post-stroke. The patients underwent transcranial magnetic stimulation to determine the presence of upper limb motor evoked potentials contralateral to the lesion, and MRI at 10–14 days to assess: FA asymmetry (ROI: posterior limb of the internal capsule), lesion load evaluated with tractography in the CST and in sensorimotor tracts. The primary upper limb motor outcome was the Action Research Arm Test, a measure of upper limb activity according to the ICF. Different prediction models were tested and the authors concluded that the PREP2 score, that includes age, SAFE and NIHSS scores as well as transcranial magnetic stimulation results, without any MRI biomarker, made correct predictions for 75% of the patients (114). DTI results were not included in the model because prediction accuracies of decision trees remained equivalent, whether or not these results were included. In order to build robust predictive models testing the magnitude of effect of different variables on upper limb motor outcomes, large samples of subjects are required.

The analysis of large sets of data, such as the ongoing ENIGMA project (http://enigma.ini.usc.edu/ongoing/enigma-stroke-recovery/) is expected to help in closing the gap in knowledge about the relevance of DTI biomarkers in research and clinical practice, to define motor prognosis. At the moment, DTI is not routinely performed in clinical practice for motor prognostication in stroke.

This study has some limitations. First, for the purpose of the review, we excluded studies not reporting metrics, such as: myelin quantification, apparent diffusion coefficients, WM volume or qualitative tractography-based information. We also excluded studies not based on the tensor, such as kurtosis or HARDI modeling, as well as microstructural-directed sequences, such as CHARMED/NODDI. All of them may convey complementary, critical information about the underlying WM alterations in the CST in stroke. Second, the choice of keywords may have led to non-inclusion of studies that addressed the aims of this review.

## Conclusions and Future Directions

FA in the CST, measured within the first hours to 3 months after stroke, has emerged as a potential DTI biomarker of motor recovery. Further research about its relevance, involving analysis of large sets of data from multiple centers, will benefit from definition of minimal standards and optimal pipelines for data acquisition, analysis, and reporting.

To perform whole-brain voxel wise and ROI analysis, according to the published studies in the field, it is suggested to: (1) acquire at least 30 non-collinear directions, as more accurate sampling reduces orientational dependence and enhances accuracy and precision of DTI metrics (10); (2) use at least 6 interspersed low *b*-value images (such as zero), reducing the risk of systematic errors due to subject motion (10); (3) use an optimal *b*-value (around 1,000 s/mm^2^), depending on the other physical parameters (28, 31, 33); (4) report parameters of acquisition employed for correction of EPI distortions (31, 115, 116); (5) whenever possible, opt for a HARDI protocol if the goal is to perform tractography. The suggested steps of pre and post-processing discussed in this review should take into consideration the limitations of the acquisition. Clear information about acquisition parameters and methodological choices of processing strategies should be provided—if necessary, due to limits in the number of words according to guidelines of different journals, as on-line supplemental material.

The decrease in methodological heterogeneity and enhancement of reproducibility will advance the field by setting the stage for large studies with good-quality data in order to define the clinical relevance of DTI in prediction of motor disability from stroke.

Finally, in the revised studies, the goal was not to test comprehensive predictive models that included DTI results. In order to determine whether DTI will have a role on prediction of motor recovery after stroke, it is necessary to test different models in large sets of data. DTI may reach a place in clinical practice if accuracy of a model is enhanced by this imaging tool, compared to models that only include variables that can be quickly and easily obtained such as bedside clinical evaluation.

## Author Contributions

AC and LM contributed to the conception and design of the study. LM wrote the first draft of the manuscript. All authors contributed to manuscript revision, read, and approved the submitted version.

### Conflict of Interest Statement

The authors declare that the research was conducted in the absence of any commercial or financial relationships that could be construed as a potential conflict of interest.
