# Supplementary material for: Diffusion Tensor Imaging Biomarkers to Predict Motor Outcomes in Stroke: A Narrative Review
Source: Front Neurol. 2019 May 8;10:445. doi: 10.3389/fneur.2019.00445 (PMC6530391; doi:10.3389/fneur.2019.00445)
Supplement: Supplementary file 1 [file Table_1.DOCX]

**Supplementary file**

**Manuscripts excluded by title**

1. Abela E, Missimer J, Wiest R, Federspiel A, Hess C, Sturzenegger M, et al. Lesions to Primary Sensory and Posterior Parietal Cortices Impair Recovery from Hand Paresis after Stroke. Plos One. 2012;7(2).

2. Alonso A, Gass A, Rossmanith C, Kern R, Griebe M, Binder J, et al. Clinical and MRI patterns of pericallosal artery infarctions: the significance of supplementary motor area lesions. J Neurol. 2012;259(5):944-51.

3. Amengual JL, Valero-Cabre A, de las Heras MV, Rojo N, Froudist-Walsh S, Ripolles P, et al. Prognostic value of cortically induced motor evoked activity by TMS in chronic stroke: Caveats from a revealing single clinical case. Bmc Neurology. 2012;12.

4. Archer DB, Misra G, Patten C, Coombes SA. Microstructural Properties of Premotor Pathways Predict Visuomotor Performance in Chronic Stroke. Human Brain Mapping. 2016;37(6):2039-54.

5. Archer DB, Vaillancourt DE, Coombes SA. A Template and Probabilistic Atlas of the Human Sensorimotor Tracts using Diffusion MRI. Cerebral Cortex. 2018;28(5):1685-99.

6. Arichi T, Counsell SJ, Allievi AG, Chew AT, Martinez-Biarge M, Mondi V, et al. The effects of hemorrhagic parenchymal infarction on the establishment of sensori-motor structural and functional connectivity in early infancy. Neuroradiology. 2014;56(11):985-94.

7. Auriat AM, Neva JL, Peters S, Ferris JK, Boyd LA. A review of transcranial magnetic stimulation and multimodal neuroimaging to characterize post-stroke neuroplasticity. Frontiers in Neurology. 2015;6.

8. Barghi A, Allendorfer JB, Taub E, Womble B, Hicks JM, Uswatte G, et al. Phase II Randomized Controlled Trial of Constraint-Induced Movement Therapy in Multiple Sclerosis. Part 2: Effect on White Matter Integrity. Neurorehabil Neural Repair. 2018;32(3):233-41.

9. Borstad AL, Bird T, Choi S, Goodman L, Schmalbrock P, Nichols-Larsen DS. Sensorimotor Training and Neural Reorganization After Stroke: A Case Series. Journal of Neurologic Physical Therapy. 2013;37(1):27-36.

10. Bradnam LV, Stinear CM, Barber PA, Byblow WD. Contralesional hemisphere control of the proximal paretic upper limb following stroke. Cereb Cortex. 2012;22(11):2662-71.

11. Brodie SM, Borich MR, Boyd LA. Impact of 5-Hz rTMS over the primary sensory cortex is related to white matter volume in individuals with chronic stroke. European Journal of Neuroscience. 2014;40(9):3405-12.

12. Byrnes TJD, Barrick TR, Bell BA, Clark CA. Semiautomatic tractography: motor pathway segmentation in patients with intracranial vascular malformations. Journal of Neurosurgery. 2009;111(1):132-40.

13. Cahill-Rowley K, Rose J. Etiology of impaired selective motor control: emerging evidence and its implications for research and treatment in cerebral palsy. Developmental Medicine and Child Neurology. 2014;56(6):522-8.

14. Caria A, Weber C, Brotz D, Ramos A, Ticini LF, Gharabaghi A, et al. Chronic stroke recovery after combined BCI training and physiotherapy: a case report. Psychophysiology. 2011;48(4):578-82.

15. Carter AR, Patel KR, Astafiev SV, Snyder AZ, Rengachary J, Strube MJ, et al. Upstream Dysfunction of Somatomotor Functional Connectivity After Corticospinal Damage in Stroke. Neurorehabilitation and Neural Repair. 2012;26(1):7-19.

16. Chang MC, Jung YJ, Jang SH. Motor recovery via transcallosal and transpontine fibers in a patient with intracerebral hemorrhage. Am J Phys Med Rehabil. 2014;93(8):708-13.

17. Chang WH, Kim YB, Ohn SH, Park CH, Kim ST, Kim YH. Double decussated ipsilateral corticospinal tract in schizencephaly. Neuroreport. 2009;20(16):1434-8.

18. Chang WH, Park CH, Kim DY, Shin YI, Ko MH, Lee A, et al. Cerebrolysin combined with rehabilitation promotes motor recovery in patients with severe motor impairment after stroke. Bmc Neurology. 2016;16.

19. Chen HY, Jiang L, Zhang H, Zhang YD, Geng W, Feng Y, et al. Corticospinal tract changes in acute brainstem ischemic stroke patients: A diffusion kurtosis imaging study. Neurology India. 2018;66(3):726-32.

20. Chenot Q, Tzourio-Mazoyer N, Rheault F, Descoteaux M, Crivello F, Zago L, et al. A population-based atlas of the human pyramidal tract in 410 healthy participants. Brain Struct Funct. 2018.

21. Cho HM, Choi BY, Chang CH, Kim SH, Lee J, Chang MC, et al. The clinical characteristics of motor function in chronic hemiparetic stroke patients with complete corticospinal tract injury. NeuroRehabilitation. 2012;31(2):207-13.

22. Choi EJ, Kyeong S, Jeon HM, Kang H, Kim DH. Brain mapping of motor and functional recovery after supratentorial stroke. Neuroreport. 2018;29(14):1217-22.

23. Choi GS, Kim OL, Kim SH, Ahn SH, Cho YW, Son SM, et al. Classification of Cause of Motor Weakness in Traumatic Brain Injury Using Diffusion Tensor Imaging. Archives of Neurology. 2012;69(3):363-7.

24. Choi TW, Jang SG, Yang SN, Pyun SB. Factors affecting the motor evoked potential responsiveness and parameters in patients with supratentorial stroke. Ann Rehabil Med. 2014;38(1):19-28.

25. Clark MG, Smallwood Shoukry R, Huang CJ, Danielian LE, Bageac D, Floeter MK. Loss of functional connectivity is an early imaging marker in primary lateral sclerosis. Amyotroph Lateral Scler Frontotemporal Degener. 2018:1-8.

26. Colon-Perez LM, Triplett W, Bohsali A, Corti M, Nguyen PT, Patten C, et al. A majority rule approach for region-of-interest-guided streamline fiber tractography. Brain Imaging and Behavior. 2016;10(4):1137-47.

27. Cuingnet R, Rosso C, Chupin M, Lehericy S, Dormont D, Benali H, et al. Spatial regularization of SVM for the detection of diffusion alterations associated with stroke outcome. Medical Image Analysis. 2011;15(5):729-37.

28. Cuingnet R, Rosso C, Lehericy S, Dormont D, Benali H, Samson Y, et al. Spatially Regularized SVM for the Detection of Brain Areas Associated with Stroke Outcome. In: Jiang T, Navab N, Pluim JPW, Viegever MA, editors. Medical Image Computing and Computer-Assisted Intervention - Miccai 2010, Pt I. Lecture Notes in Computer Science. 63612010. p. 316-+.

29. Cunningham DA, Machado A, Janini D, Varnerin N, Bonnett C, Yue G, et al. Assessment of Inter-Hemispheric Imbalance Using Imaging and Noninvasive Brain Stimulation in Patients With Chronic Stroke. Archives of Physical Medicine and Rehabilitation. 2015;96(4):S94-S103.

30. Danielian LE, Iwata NK, Thomasson DM, Floeter MK. Reliability of fiber tracking measurements in diffusion tensor imaging for longitudinal study. Neuroimage. 2010;49(2):1572-80.

31. Darwazeh R, Wei M, Zhong JJ, Zhang ZS, Lv FJ, Darwazeh M, et al. Significant Injury of the Mammillothalamic Tract without Injury of the Corticospinal Tract After Aneurysmal Subarachnoid Hemorrhage: A Retrospective Diffusion Tensor Imaging Study. World Neurosurgery. 2018;114:E624-E30.

32. Dawes H, Enzinger C, Johansen-Berg H, Bogdanovic M, Guy C, Collett J, et al. Walking performance and its recovery in chronic stroke in relation to extent of lesion overlap with the descending motor tract. Experimental Brain Research. 2008;186(2):325-33.

33. DeVetten G, Coutts SB, Hill MD, Goyal M, Eesa M, O'Brien B, et al. Acute Corticospinal Tract Wallerian Degeneration Is Associated With Stroke Outcome. Stroke. 2010;41(4):751-6.

34. Dinomais M, Hertz-Pannier L, Groeschel S, Chabrier S, Delion M, Husson B, et al. Long term motor function after neonatal stroke: Lesion localization above all. Hum Brain Mapp. 2015;36(12):4793-807.

35. Do KH, Yeo SS, Lee J, Jang SH. Injury of the corticoreticular pathway in patients with proximal weakness following cerebral infarct: Diffusion tensor tractography study. Neuroscience Letters. 2013;546:21-5.

36. Domi T, deVeber G, Shroff M, Kouzmitcheva E, MacGregor DL, Kirton A. Corticospinal Tract Pre-Wallerian Degeneration A Novel Outcome Predictor for Pediatric Stroke on Acute MRI. Stroke. 2009;40(3):780-7.

37. Domin M, Langner S, Hosten N, Lotze M. Comparison of Parameter Threshold Combinations for Diffusion Tensor Tractography in Chronic Stroke Patients and Healthy Subjects. Plos One. 2014;9(5).

38. Du J, Yang F, Zhang ZQ, Hu JZ, Xu Q, Hu JP, et al. Early functional MRI activation predicts motor outcome after ischemic stroke: a longitudinal, multimodal study. Brain Imaging and Behavior. 2018;12(6):1804-13.

39. Eliassen JC, Boespflug EL, Lamy M, Allendorfer J, Chu WJ, Szaflarski JP. Brain-Mapping Techniques for Evaluating Poststroke Recovery and Rehabilitation: A Review. Topics in Stroke Rehabilitation. 2008;15(5):427-50.

40. Ellis MJ, Rutka JT, Kulkarni AV, Dirks PB, Widjaja E. Corticospinal tract mapping in children with ruptured arteriovenous malformations using functionally guided diffusion-tensor imaging Report of 3 cases. Journal of Neurosurgery-Pediatrics. 2012;9(5):505-10.

41. Ferris JK, Edwards JD, Ma JA, Boyd LA. Changes to White Matter Microstructure in Transient Ischemic Attack: A Longitudinal Diffusion Tensor Imaging Study. Human Brain Mapping. 2017;38(11):5795-803.

42. Floeter MK, Danielian LE, Braun LE, Wu T. Longitudinal diffusion imaging across the C9orf72 clinical spectrum. J Neurol Neurosurg Psychiatry. 2018;89(1):53-60.

43. Floeter MK, Katipally R, Kim MP, Schanz O, Stephen M, Danielian L, et al. Impaired corticopontocerebellar tracts underlie pseudobulbar affect in motor neuron disorders. Neurology. 2014;83(7):620-7.

44. Frost KL, Carey JR, Broback TW, Carlson NL, Daggett CA, Dalbec MM, et al. N-of-1 Trial in Person with Pontine Stroke Receiving Repetitive Transcranial Magnetic Stimulation to Improve Hand Function. J Neuroimaging Psychiatry Neurol. 2017;2(2):36-42.

45. Gao J, Li XJ, Hou X, Ding A, Chan KC, Sun QL, et al. TRACT-BASED SPATIAL STATISTICS (TBSS): APPLICATION TO DETECTING WHITE MATTER TRACT VARIATION IN MILD HYPOXIC-ISCHEMIC NEONATES. 2012 Annual International Conference of the Ieee Engineering in Medicine and Biology Society. IEEE Engineering in Medicine and Biology Society Conference Proceedings2012. p. 432-5.

46. George E, Heier L, Kovanlikaya I, Greenfield J. Diffusion tensor imaging of pyramidal tract reorganization after pediatric stroke. Childs Nervous System. 2014;30(6):1135-9.

47. Globas C, Lam JM, Zhang WH, Imanbayev A, Hertler B, Becker C, et al. Mesencephalic Corticospinal Atrophy Predicts Baseline Deficit but Not Response to Unilateral or Bilateral Arm Training in Chronic Stroke. Neurorehabilitation and Neural Repair. 2011;25(1):81-7.

48. Gold MM, Shifteh K, Valdberg S, Lombard J, Lipton ML. Brain injury due to ventricular shunt placement delineated by diffusion tensor imaging (DTI) tractography. Neurologist. 2008;14(4):252-4.

49. Gordon AL, Wood A, Tournier JD, Hunt RW. Corticospinal tract integrity and motor function following neonatal stroke: a case study. Bmc Neurology. 2012;12.

50. Goto T, Saitoh Y, Hashimoto N, Hirata M, Kishima H, Oshino S, et al. Diffusion tensor fiber tracking in patients with central post-stroke pain; correlation with efficacy of repetitive transcranial magnetic stimulation. Pain. 2008;140(3):509-18.

51. Groeschel S, Hertz-Pannier L, Delion M, Loustau S, Husson B, Kossorotoff M, et al. Association of transcallosal motor fibres with function of both hands after unilateral neonatal arterial ischemic stroke. Developmental Medicine and Child Neurology. 2017;59(10):1042-8.

52. Guo ZW, Jin Y, Peng HT, Xing GQ, Liao X, Wang YF, et al. Ipsilesional High Frequency Repetitive Transcranial Magnetic Stimulation Add-On Therapy Improved Diffusion Parameters of Stroke Patients with Motor Dysfunction: A Preliminary DTI Study. Neural Plasticity. 2016.

53. Habegger S, Wiest R, Wader BJ, Mordasini P, Gralla J, Hani L, et al. Relating Acute Lesion Loads to Chronic Outcome in Ischemic Stroke-An Exploratory Comparison of Mismatch Patterns and Predictive Modeling. Frontiers in Neurology. 2018;9.

54. Han BS, Ahn SH, Jang SH. Cortical reorganization demonstrated by diffusion tensor tractography analyzed using functional MRI activation. Neurorehabilitation. 2008;23(2):171-4.

55. Han BS, Hong JH, Hong C, Yeo SS, Lee DH, Cho HK, et al. Location of the corticospinal tract at the corona radiata in human brain. Brain Research. 2010;1326:75-80.

56. Hao XZ, Yin LK, Tian JQ, Li CC, Feng XY, Yao ZW, et al. Inhibition of Notch1 Signaling at the Subacute Stage of Stroke Promotes Endogenous Neurogenesis and Motor Recovery After Stroke. Frontiers in Cellular Neuroscience. 2018;12.

57. Hayward KS, Brauer SG, Ruddy KL, Lloyd D, Carson RG. Repetitive reaching training combined with transcranial Random Noise Stimulation in stroke survivors with chronic and severe arm paresis is feasible: a pilot, triple-blind, randomised case series. Journal of Neuroengineering and Rehabilitation. 2017;14.

58. Hayward KS, Neva JL, Mang CS, Peters S, Wadden KP, Ferris JK, et al. Interhemispheric Pathways Are Important for Motor Outcome in Individuals with Chronic and Severe Upper Limb Impairment Post Stroke. Neural Plasticity. 2017.

59. Hedna VS, Jain S, Rabbani O, Nadeau SE. Mechanisms of arm paresis in middle cerebral artery distribution stroke: Pilot study. Journal of Rehabilitation Research and Development. 2013;50(8):1113-21.

60. Helton KJ, Weeks JK, Phillips NS, Zou P, Kun LE, Khan RB, et al. Diffusion tensor imaging of brainstem tumors: axonal degeneration of motor and sensory tracts. Journal of Neurosurgery-Pediatrics. 2008;1(4):270-6.

61. Hiraga A, Uzawa A, Tanaka S, Ogawara K, Kamitsukasa I. Pure monoparesis of the leg due to cerebral infarctions: A diffusion-weighted imaging study. Journal of Clinical Neuroscience. 2009;16(11):1414-6.

62. Hodge J, Goodyear B, Carlson H, Wei XC, Kirton A. Segmental Diffusion Properties of the Corticospinal Tract and Motor Outcome in Hemiparetic Children With Perinatal Stroke. Journal of Child Neurology. 2017;32(6):550-9.

63. Hong JH, Jang SH. Is combined functional magnetic resonance imaging and diffusion tensor tractography a useful tool for evaluation of somatosensory dysfunction recovery after intracerebral hemorrhage? Neural Regeneration Research. 2010;5(14):1109-12.

64. Hong JH, Jang SH. Peri-infarct reorganization at the centrum semiovale and corona radiata A diffusion tensor tractography and functional MRI study. Neural Regeneration Research. 2011;6(11):826-9.

65. Hong JH, Kim SH, Kim OL, Byun WM, Jang SH. Neural tract injuries by brain herniations after head trauma. J Head Trauma Rehabil. 2012;27(2):154-8.

66. Hong JH, Lee J, Cho YW, Byun WM, Cho HK, Son SM, et al. Limb apraxia in a patient with cerebral infarct: Diffusion tensor tractography study. Neurorehabilitation. 2012;30(4):255-9.

67. Hong JH, Son SM, Byun WM, Jang HW, Ahn SH, Jang SH. Aberrant pyramidal tract in medial lemniscus of brainstem in the human brain. Neuroreport. 2009;20(7):695-7.

68. Hong X, Lu ZK, Teh I, Nasrallah FA, Teo WP, Ang KK, et al. Brain plasticity following MI-BCI training combined with tDCS in a randomized trial in chronic subcortical stroke subjects: a preliminary study. Sci Rep. 2017;7(1):9222.

69. Hsieh CT, Chen CY, Chiang YH, Chang CH, Chang CF. Role of diffusion tensor imaging in a patient with spontaneous intracerebral hematoma treated by stereotactic evacuation. Surgical Neurology. 2008;70(1):75-8.

70. Hu J, Li C, Hua Y, Zhang B, Gao BY, Liu PL, et al. Constrained-induced movement therapy promotes motor function recovery by enhancing the remodeling of ipsilesional corticospinal tract in rats after stroke. Brain Res. 2019;1708:27-35.

71. Huang YC, Tsai YH, Lee JD, Weng HH, Lin LC, Lin YH, et al. Hemodynamic Factors May Play a Critical Role in Neurological Deterioration Occurring within 72 hrs after Lacunar Stroke. Plos One. 2014;9(10).

72. Hunter SM, Johansen-Berg H, Ward N, Kennedy NC, Chandler E, Weir CJ, et al. Functional Strength Training and Movement Performance Therapy for Upper Limb Recovery Early Poststroke-Efficacy, Neural Correlates, Predictive Markers, and Cost-Effectiveness: FAST-INdiCATE Trial. Frontiers in Neurology. 2018;8.

73. Husson B, Durand C, Hertz-Pannier L. [Recommendations for imaging neonatal ischemic stroke]. Arch Pediatr. 2017;24(9s):9s19-9s27.

74. Husson B, Hertz-Pannier L, Renaud C, Allard D, Presles E, Landrieu P, et al. Motor outcomes after neonatal arterial ischemic stroke related to early MRI data in a prospective study. Pediatrics. 2010;126(4):912-8.

75. Iwata NK, Kwan JY, Danielian LE, Butman JA, Tovar-Moll F, Bayat E, et al. White matter alterations differ in primary lateral sclerosis and amyotrophic lateral sclerosis. Brain. 2011;134(Pt 9):2642-55.

76. Jang SG, Pyun SB. Diffusion tensor tractography in two cases of kernohan-woltman notch phenomenon. Ann Rehabil Med. 2013;37(6):879-85.

77. Jang SH. A review of corticospinal tract location at corona radiata and posterior limb of the internal capsule in human brain. Neurorehabilitation. 2009;24(3):279-83.

78. Jang SH. A review of the ipsilateral motor pathway as a recovery mechanism in patients with stroke. Neurorehabilitation. 2009;24(4):315-20.

79. Jang SH. Medial reorganization of motor function in corona radiata following middle cerebral artery infarction A case report. Neural Regeneration Research. 2009;4(9):668-71.

80. Jang SH. The role of the corticospinal tract in motor recovery in patients with a stroke: A review. Neurorehabilitation. 2009;24(3):285-90.

81. Jang SH. Perilesional reorganization of motor function in stroke patients. Neural Regeneration Research. 2010;5(21):1668-72.

82. Jang SH. Prediction of motor outcome for hemiparetic stroke patients using diffusion tensor imaging: A review. Neurorehabilitation. 2010;27(4):367-72.

83. Jang SH. Spared integrity of corticospinal tract within a pontine infarct A diffusion tensor tractography study. Neural Regeneration Research. 2010;5(20):1552-4.

84. Jang SH. The recovery of walking in stroke patients: a review. Int J Rehabil Res. 2010;33(4):285-9.

85. Jang SH. Transcallosal motor pathway from affected motor cortex to affected hand in a patient with corona radiata infarct A diffusion tensor tractography and transcranial magnetic stimulation study. Neural Regeneration Research. 2010;5(14):1117-20.

86. Jang SH. Unusual long-term motor recovery in a patient with corona radiata infarct. Neural Regeneration Research. 2010;5(17):1353-6.

87. Jang SH. A review of diffusion tensor imaging studies on motor recovery mechanisms in stroke patients. Neurorehabilitation. 2011;28(4):345-52.

88. Jang SH. Diffusion tensor imaging studies on corticospinal tract injury following traumatic brain injury: A review. Neurorehabilitation. 2011;29(4):339-45.

89. Jang SH. Isolated corticospinal tract in a patient with intracerebral hemorrhage A diffusion tensor tractography and transcranial magnetic stimulation study. Neural Regeneration Research. 2011;6(7):558-60.

90. Jang SH. Somatotopic Arrangement and Location of the Corticospinal Tract in the Brainstem of the Human Brain. Yonsei Medical Journal. 2011;52(4):553-7.

91. Jang SH. Motor outcome and motor recovery mechanisms in pontine infarct: A review. Neurorehabilitation. 2012;30(2):147-52.

92. Jang SH. Motor Recovery Mechanisms in Patients with Middle Cerebral Artery Infarct: A Mini-Review. European Neurology. 2012;68(4):234-9.

93. Jang SH. Motor function-related maladaptive plasticity in stroke: A review. Neurorehabilitation. 2013;32(2):311-6.

94. Jang SH. Motor recovery by improvement of limb-kinetic apraxia in a chronic stroke patient. NeuroRehabilitation. 2013;33(2):195-200.

95. Jang SH. THE CORTICOSPINAL TRACT FROM THE VIEWPOINT OF BRAIN REHABILITATION. Journal of Rehabilitation Medicine. 2014;46(3):193-9.

96. Jang SH, Chang CH, Lee HD. Limb-Kinetic Apraxia Due to Injury of the Corticofugal Tract from the Secondary Motor Area in a Stroke Patient. Am J Phys Med Rehabil. 2016;95(7):e115-6.

97. Jang SH, Chang CH, Lee J, Kim CS, Seo JP, Yeo SS. Functional Role of the Corticoreticular Pathway in Chronic Stroke Patients. Stroke. 2013;44(4):1099-104.

98. Jang SH, Chang MC. Recovery of an injured corticoreticulospinal tract in a patient with pontine hemorrhage. Int J Stroke. 11. United States2016. p. Np18-9.

99. Jang SH, Choi BY, Chang CH, Kim SH, Chang MC. Prediction of motor outcome based on diffusion tensor tractography findings in thalamic hemorrhage. International Journal of Neuroscience. 2013;123(4):233-9.

100. Jang SH, Choi BY, Kim SH, Chang CLH, Jung YJ, Yeo SS. INJURY OF THE CORTICORETICULAR PATHWAY IN SUBARACHNOID HAEMORRHAGE AFTER RUPTURE OF A CEREBRAL ARTERY ANEURYSM. Journal of Rehabilitation Medicine. 2015;47(2):133-7.

101. Jang SH, Hong JH, Ahn SH, Son SM, Cho YW. Motor function reorganization in a patient with a brainstem lesion: DTT, fMRI and TMS study. Neurorehabilitation. 2010;26(2):167-71.

102. Jang SH, Kim DH, Kim SH, Seo JP. The relation between the motor evoked potential and diffusion tensor tractography for the corticospinal tract in chronic hemiparetic patients with cerebral infarct. Somatosens Mot Res. 2017;34(2):134-8.

103. Jang SH, Kim DS, Son SM, Cho YW, Kim SH, Kim OL, et al. Clinical application of diffusion tensor tractography for elucidation of the causes of motor weakness in patients with traumatic brain injury. Neurorehabilitation. 2009;24(3):273-8.

104. Jang SH, Kim SH, Seo JP. Recovery of an injured corticofugal tract from the supplementary motor area in a patient with traumatic brain injury A case report. Medicine. 2018;97(7).

105. Jang SH, Kwon HG. Deterioration of pre-existing hemiparesis due to injury of the ipsilateral anterior corticospinal tract. Bmc Neurology. 2013;13.

106. Jang SH, Kwon HG. Change of Neural Connectivity of the Red Nucleus in Patients with Striatocapsular Hemorrhage: A Diffusion Tensor Tractography Study. Neural Plasticity. 2015.

107. Jang SH, Kwon HG. Change of the anterior corticospinal tract on the normal side of the brain in chronic stroke patients: Diffusion tensor imaging study. Somatosensory and Motor Research. 2015;32(1):25-30.

108. Jang SH, Kwon HG. Delayed gait recovery with recovery of an injured corticoreticulospinal tract in a chronic hemiparetic patient A case report. Medicine. 2016;95(46).

109. Jang SH, Kwon HG. Delayed regaining of gait ability in a patient with brain injury: A case report. Medicine. 2016;95(38).

110. Jang SH, Kwon HG. Recovery of an injured corticospinal tract during the early stage of rehabilitation following pontine infarction. Neural Regeneration Research. 2016;11(3):519-20.

111. Jang SH, Kwon YH, Lee MY, Lee DY, Hong JH. Difference of neural connectivity for motor function in chronic hemiparetic stroke patients with intracerebral hemorrhage. Neuroscience Letters. 2012;531(2):80-5.

112. Jang SH, Lee HD. Delayed recovery of the affected finger extensors at chronic stage in a stroke patient: A case report. Medicine (Baltimore). 2017;96(43):e8023.

113. Jang SH, Lee J, Lee HD. Peri-infarct reorganization of an injured corticoreticulospinal tract in a patient with cerebral infarct. Int J Stroke. 2015;10(6):E62-3.

114. Jang SH, Lee J, Seo JP. Reorganization of the corticospinal tract to anterior area of corona radiata infarct. Int J Stroke. 2015;10(7):E76-7.

115. Jang SH, Park KA, Ahn SH, Cho YW, Byun WM, Son SM, et al. Transcallosal fibers from corticospinal tract in patients with cerebral infarct. Neurorehabilitation. 2009;24(2):159-64.

116. Jang SH, Sea JP. Differences of the medial lemniscus and spinothalamic tract according to the cortical termination areas: A diffusion tensor tractography study. Somatosensory and Motor Research. 2015;32(2):67-71.

117. Jang SH, Seo JP. The distribution of the cortical origin of the corticoreticular pathway in the human brain: A diffusion tensor imaging study. Somatosensory and Motor Research. 2014;31(4):204-8.

118. Jang SH, Seo JP. Aging of corticospinal tract fibers according to the cerebral origin in the human brain: A diffusion tensor imaging study. Neuroscience Letters. 2015;585:77-81.

119. Jang SH, Seo JP. Limb-kinetic apraxia due to injury of corticofugal tracts from secondary motor area in patients with corona radiata infarct. Acta Neurol Belg. 2016;116(4):467-72.

120. Jang SH, Seo YS. Injuries of neural tracts in a patient with CADASIL: a diffusion tensor imaging study. Bmc Neurology. 2015;15.

121. Jang SH, Seo YS. Effect of Neuromuscular Electrical Stimulation Training on the Finger Extensor Muscles for the Contralateral Corticospinal Tract in Normal Subjects: A Diffusion Tensor Tractography Study. Frontiers in Human Neuroscience. 2018;12.

122. Jang SH, Yang DS, Lee J. Preservation of the integrity of the corticospinal tract in a patient with medulla infarct. Am J Phys Med Rehabil. 2009;88(3):256-8.

123. Jang SH, Yeo SS. Recovery of an injured corticoreticular pathway via transcallosal fibers in a patient with intracerebral hemorrhage. Bmc Neurology. 2014;14.

124. Jang SH, Yi JH, Chang CH, Jung YJ, Kim SH, Lee J, et al. Prediction of motor outcome by shoulder subluxation at early stage of stroke. Medicine. 2016;95(32).

125. Jang SH, Yi JH, Choi BY, Chang CH, Jung YJ, Do Lee H, et al. Changes of the corticospinal tract in the unaffected hemisphere in stroke patients: A diffusion tensor imaging study. Somatosensory and Motor Research. 2016;33(1):1-7.

126. Jang S, Kim J, Seo Y, Kwak S. Recovery of an injured corticobulbar tract in a patient with stroke: A case report. Medicine (Baltimore). 2017;96(38):e7636.

127. Jayaram G, Stagg CJ, Esser P, Kischka U, Stinear J, Johansen-Berg H. Relationships between functional and structural corticospinal tract integrity and walking post stroke. Clin Neurophysiol. 2012;123(12):2422-8.

128. Jiang L, Xu HJ, Yu CS. Brain Connectivity Plasticity in the Motor Network after Ischemic Stroke. Neural Plasticity. 2013.

129. Jiao Y, Lin F, Wu J, Li H, Chen X, Li Z, et al. Brain Arteriovenous Malformations Located in Premotor Cortex: Surgical Outcomes and Risk Factors for Postoperative Neurologic Deficits. World Neurosurg. 2017;105:432-40.

130. Jiao Y, Lin F, Wu J, Li H, Chen X, Li Z, et al. Brain Arteriovenous Malformations Supplied by the Anterior Choroidal Artery: Treatment Outcomes and Risk Factors for Worsened Muscle Strength After Surgical Resection. World Neurosurg. 2017;104:567-74.

131. Jo HM, Choi BY, Chang CH, Kim SH, Lee J, Chang MC, et al. The clinical characteristics of motor function in chronic hemiparetic stroke patients with complete corticospinal tract injury. Neurorehabilitation. 2012;31(2):207-13.

132. Jones PS, Pomeroy VM, Wang J, Schlaug G, Marrapu ST, Geva S, et al. Does Stroke Location Predict Walk Speed Response to Gait Rehabilitation? Human Brain Mapping. 2016;37(2):689-703.

133. Jung YJ, Jang SH, Yeo SS, Lee E, Kim S, Lee DG, et al. Medial Lemniscus Lesion in Pediatric Hemiplegic Patients without Corticospinal Tract and Posterior Thalamic Radiation Lesion. European Neurology. 2012;67(4):211-6.

134. Kalinosky BT, Schindler-Ivens S, Schmit BD. White matter structural connectivity is associated with sensorimotor function in stroke survivors. Neuroimage-Clinical. 2013;2:767-81.

135. Kamson DO, Juhasz C, Shin J, Behen ME, Guy WC, Chugani HT, et al. Patterns of Structural Reorganization of the Corticospinal Tract in Children With Sturge-Weber Syndrome. Pediatric Neurology. 2014;50(4):337-42.

136. Kaseka ML, Moharir M, deVeber G, MacGregor D, Askalan R, Dlamini N. Prognostication Value of Descending Corticospinal Tract DWI Signal in Neonatal Cerebral Sinovenous Thrombosis. Pediatric Neurology. 2016;59:90-4.

137. Kasprian G, Seidel S. [Modern neuroimaging of brain plasticity]. Radiologe. 2010;50(2):136-43.

138. Kato H, Izumiyama M. IMPAIRED MOTOR CONTROL DUE TO PROPRIOCEPTIVE SENSORY LOSS IN A PATIENT WITH CEREBRAL INFARCTION LOCALIZED TO THE POSTCENTRAL GYRUS. Journal of Rehabilitation Medicine. 2015;47(2):187-90.

139. Kato H, Takeda T, Ohara K, Tei H, Nishizawa E. Rostrocaudal Thickness on Sagittal Diffusion-weighted Imaging as a Predictor of Motor Deficits in an Acute Isolated Pontine Infarction. Journal of Stroke & Cerebrovascular Diseases. 2015;24(3):622-8.

140. Keser Z, Yozbatiran N, Francisco GE, Hasan KM. A note on the mapping and quantification of the human brain corticospinal tract. European Journal of Radiology. 2014;83(9):1703-5.

141. Kidokoro H, Kubota T, Ohe H, Hattori T, Kato Y, Miyajima Y, et al. Diffusion-weighted Magnetic Resonance Imaging in Infants with Periventricular Leukomalacia. Neuropediatrics. 2008;39(4):233-8.

142. Kim BR, Moon WJ, Kim H, Jung E, Lee J. Association of Dysphagia With Supratentorial Lesions in Patients With Middle Cerebral Artery Stroke. Annals of Rehabilitation Medicine-Arm. 2016;40(4):637-46.

143. Kim BR, Moon WJ, Kim H, Jung E, Lee J. Transcranial Magnetic Stimulation and Diffusion Tensor Tractography for Evaluating Ambulation after Stroke. Journal of Stroke. 2016;18(2):220-6.

144. Kim DG, Kim SH, Kim OL, Cho YW, Son SM, Jang SH. Long-term recovery of motor function in a quadriplegic patient with diffuse axonal injury and traumatic hemorrhage: A case report. Neurorehabilitation. 2009;25(2):117-22.

145. Kim DH, Kyeong S, Cho Y, Jung TM, Ahn SJ, Park YG. Usefulness of voxel-based lesion mapping for predicting motor recovery in subjects with basal ganglia hemorrhage A preliminary study with 2 case reports. Medicine. 2016;95(23).

146. Kim EJ, Park CH, Chang WH, Lee A, Kim ST, Shin YI, et al. The brain-derived neurotrophic factor Val66Met polymorphism and degeneration of the corticospinal tract after stroke: a diffusion tensor imaging study. European Journal of Neurology. 2016;23(1):76-84.

147. Kim HS, Park JW, Bai DS, Jeong JY, Hong JH, Son SM, et al. Diffusion tensor imaging findings in neurologically asymptomatic patients with end stage renal disease. Neurorehabilitation. 2011;29(1):111-6.

148. Kim HW, Lee DG. Resting-State Metabolism of Hand Knob Area on F-18-FDG PET-CT According to Hand Function and Tractography of Corticospinal Tract After Stroke. Annals of Rehabilitation Medicine-Arm. 2017;41(2):171-7.

149. Kim JH, Kwon YM, Son SM. Motor function outcomes of pediatric patients with hemiplegic cerebral palsy after rehabilitation treatment: a diffusion tensor imaging study. Neural Regeneration Research. 2015;10(4):624-30.

150. Kim JH, Son SM. Activation of less affected corticospinal tract and poor motor outcome in hemiplegic pediatric patients: a diffusion tensor tractography imaging study. Neural Regeneration Research. 2015;10(12):2054-9.

151. Kim KH, Kim YH, Kim MS, Park CH, Lee A, Chang WH. Prediction of Motor Recovery Using Diffusion Tensor Tractography in Supratentorial Stroke Patients With Severe Motor Involvement. Annals of Rehabilitation Medicine-Arm. 2015;39(4):570-6.

152. Kim SH, Jang SH, Lee E, Kim S, Cho YW, Hong JH, et al. Usefulness of diffusion tensor imaging in patients who showed sustained unexplainable clinical symptom of torticollis. Neuroscience Letters. 2012;522(1):25-9.

153. Kim YB, Kalthoff D, Po C, Wiedermann D, Hoehn M. Connectivity of thalamo-cortical pathway in rat brain: combined diffusion spectrum imaging and functional MRI at 11.7 T. Nmr in Biomedicine. 2012;25(7):943-52.

154. Kim YH, Kim DS, Hong JH, Park CH, Hua N, Bickart KC, et al. Corticospinal tract location in internal capsule of human brain: diffusion tensor tractography and functional MRI study. Neuroreport. 2008;19(8):817-20.

155. Kim Y, Kim SH, Kim JS, Hong BY. Modification of Cerebellar Afferent Pathway in the Subacute Phase of Stroke. Journal of Stroke & Cerebrovascular Diseases. 2018;27(9):2445-52.

156. Kirton A, Williams E, Dowling M, Mah S, Hodge J, Carlson H, et al. Diffusion imaging of cerebral diaschisis in childhood arterial ischemic stroke. International Journal of Stroke. 2016;11(9):1028-35.

157. Koch P, Schulz R, Hummel FC. Structural connectivity analyses in motor recovery research after stroke. Annals of Clinical and Translational Neurology. 2016;3(3):233-44.

158. Koga T, Shin M, Maruyama K, Kamada K, Ota T, Itoh D, et al. Integration of corticospinal tractography reduces motor complications after radiosurgery. Int J Radiat Oncol Biol Phys. 2012;83(1):129-33.

159. Koh CL, Tang PF, Chen HI, Hsu YC, Hsieh CL, Tseng WYI. Impaired Callosal Motor Fiber Integrity and Upper Extremity Motor Impairment Are Associated With Stroke Lesion Location. Neurorehabilitation and Neural Repair. 2018;32(6-7):602-12.

160. Konomi T, Fujiyoshi K, Hikishima K, Komaki Y, Tsuji O, Okano HJ, et al. Conditions for quantitative evaluation of injured spinal cord by in vivo diffusion tensor imaging and tractography: preclinical longitudinal study in common marmosets. Neuroimage. 2012;63(4):1841-53.

161. Kou N, Park CH, Seghier ML, Leff AP, Ward NS. Can fully automated detection of corticospinal tract damage be used in stroke patients? Neurology. 2013;80(24):2242-5.

162. Koyama T, Domen K. Diffusion Tensor Fractional Anisotropy in the Superior Longitudinal Fasciculus Correlates with Functional Independence Measure Cognition Scores in Patients with Cerebral Infarction. Journal of Stroke & Cerebrovascular Diseases. 2017;26(8):1704-11.

163. Koyama T, Uchiyama Y, Domen K. Associations of Diffusion-Tensor Fractional Anisotropy and FIM Outcome Assessments After Intracerebral Hemorrhage. Journal of Stroke & Cerebrovascular Diseases. 2018;27(10):2869-76.

164. Ku J, Mendelsohn D, Chew J, Shewchuk J, Dong C, Akagami R. Ipsilateral Motor Innervation Discovered Incidentally on Intraoperative Monitoring: A Case Report. Neurosurgery. 2017;80(3):E194-E200.

165. Kuczynski AM, Dukelow SP, Hodge JA, Carlson HL, Lebel C, Semrau JA, et al. Corticospinal tract diffusion properties and robotic visually guided reaching in children with hemiparetic cerebral palsy. Human Brain Mapping. 2018;39(3):1130-44.

166. Kulikova SP, Nikulin VV, Dobrynina LA, Nazarova MA. A possible Sensory interpretation of Alternate Motor Fibers Relating to Structural Reserve during Stroke Recovery. Frontiers in Neurology. 2017;8.

167. Kumar P, Yadav AK, Misra S, Kumar A, Chakravarty K, Prasad K. Prediction of upper extremity motor recovery after subacute intracerebral hemorrhage through diffusion tensor imaging: a systematic review and meta-analysis. Neuroradiology. 2016;58(10):1043-50.

168. Kwah LK, Herbert RD. Prediction of Walking and Arm Recovery after Stroke: A Critical Review. Brain Sciences. 2016;6(4).

169. Kwak SY, Son SM, Choi BY, Chang CH, Byun WM, Kim SH, et al. Prognostic factors for motor outcome in patients with compressed corticospinal tract by intracerebral hematoma. Neurorehabilitation. 2011;29(1):85-90.

170. Kwak SY, Yeo SS, Choi BY, Chang CH, Jang SH. Corticospinal Tract Change in the Unaffected Hemisphere at the Early Stage of Intracerebral Hemorrhage: A Diffusion Tensor Tractography Study. European Neurology. 2010;63(3):149-53.

171. Kwan JY, Meoded A, Danielian LE, Wu T, Floeter MK. Structural imaging differences and longitudinal changes in primary lateral sclerosis and amyotrophic lateral sclerosis. Neuroimage Clin. 2012;2:151-60.

172. Kwon HG, Choi BY, Chang CH, Kim SH, Jung YJ, Jang SH. Recovery of an injured corticospinal tract during a critical period in a patient with intracerebral hemorrhage. Neurorehabilitation. 2013;32(1):27-32.

173. Kwon HG, Hong JH, Lee MY, Kwon YH, Jang SH. Somatotopic Arrangement of the Corticospinal Tract at the Medullary Pyramid in the Human Brain. European Neurology. 2011;65(1):46-9.

174. Kwon HG, Jang SH. Change in connection between corticospinal tract and Broca's area during motor recovery in a patient with an intracerebral hemorrhage. Neural Regeneration Research. 2011;6(17):1313-5.

175. Kwon HG, Jang SH. Compression of the bilateral corticospinal tracts by bilateral pontine hemorrhage A diffusion tensor tractography study. Neural Regeneration Research. 2011;6(4):317-20.

176. Kwon HG, Jang SH. Motor recovery in a patient with an infarct in the medullary pyramid via the corticospinal tract passing through the small spared area within the infarcted medullary pyramid. Neural Regeneration Research. 2011;6(15):1185-8.

177. Kwon HG, Jang SH. Motor recovery mechanism in a quadriplegic patient with locked-in syndrome. Neurorehabilitation. 2012;30(2):113-7.

178. Kwon HG, Jang SH. SIGNIFICANCE OF REHABILITATIVE MANAGEMENT DURING THE CRITICAL PERIOD FOR MOTOR RECOVERY IN INTRACEREBRAL HEMORRHAGE: A CASE REPORT. Journal of Rehabilitation Medicine. 2012;44(3):280-4.

179. Kwon HG, Lee DG, Choi BY, Chang CH, Kim SH, Jang SH. Recovery of the corticospinal tract after injury by transtentorial herniation: A case report. Neurorehabilitation. 2011;29(3):243-6.

180. Kwon HG, Lee DG, Son SM, Byun WM, Hong CP, Lee DH, et al. Identification of the anterior corticospinal tract in the human brain using diffusion tensor imaging. Neuroscience Letters. 2011;505(3):238-41.

181. Kwon HG, Yang JH, Park JB, Kim MH, Choi SH, Yang DS. Anatomical location and somatotopic organization of the corticospinal tract in the corona radiata of the normal human brain: a diffusion tensor tractography study. Neuroreport. 2014;25(9):710-4.

182. Kwon H, Jang SH. Delayed recovery of gait function in a patient with intracerebral haemorrhage. J Rehabil Med. 2012;44(4):378-80.

183. Kwon JY, Chang WH, Chang HJ, Yi SH, Kim MY, Kim EH, et al. Changes in diffusion tensor tractographic findings associated with constraint-induced movement therapy in young children with cerebral palsy. Clin Neurophysiol. 2014;125(12):2397-403.

184. Kwon YM, Rose J, Kim AR, Son SM. Corticoreticular tract lesion in children with developmental delay presenting with gait dysfunction and trunk instability. Neural Regeneration Research. 2017;12(9):1465-71.

185. Lakhani B, Hayward KS, Boyd LA. Hemispheric asymmetry in myelin after stroke is related to motor impairment and function. Neuroimage Clin. 2017;14:344-53.

186. Lazaridou A, Astrakas L, Mintzopoulos D, Khanicheh A, Singhal AB, Moskowitz MA, et al. Diffusion tensor and volumetric magnetic resonance imaging using an MR-compatible hand-induced robotic device suggests training-induced neuroplasticity in patients with chronic stroke. International Journal of Molecular Medicine. 2013;32(5):995-1000.

187. Lee AY, Choi BY, Kim SH, Chang CH, Jung YJ, Jang SH. Difference of injury of the corticospinal tract according to surgical or conservative treatment in patients with putaminal hemorrhage. International Journal of Neuroscience. 2016;126(5):429-35.

188. Lee DH, Hong C, Han BS. Diffusion-tensor magnetic resonance imaging for hand and foot fibers location at the corona radiata: comparison with two lesion studies. Frontiers in Human Neuroscience. 2014;8.

189. Lee DH, Lee DW, Han BS. Topographic organization of motor fibre tracts in the human brain: findings in multiple locations using magnetic resonance diffusion tensor tractography. European Radiology. 2016;26(6):1751-9.

190. Lee DH, Park JW, Park SH, Hong C. Have You Ever Seen the Impact of Crossing Fiber in DTI?: Demonstration of the Corticospinal Tract Pathway. Plos One. 2015;10(7).

191. Lee SY, Kang SH, Kim DK, Seo KM, Ro HJ, Kim JK. Changes in the corticospinal tract after wearing prosthesis in bilateral transtibial amputation. Prosthetics and Orthotics International. 2017;41(5):507-11.

192. Li JJ, Chen XL, Zhang JS, Zheng G, Lv XM, Li FY, et al. Intraoperative diffusion tensor imaging predicts the recovery of motor dysfunction after insular lesions. Neural Regeneration Research. 2013;8(15):1400-9.

193. Li J, Zuo ZT, Zhang XW, Shao XL, Lu J, Xue R, et al. Excitatory Repetitive Transcranial Magnetic Stimulation Induces Contralesional Cortico-Cerebellar Pathways After Acute Ischemic Stroke: A Preliminary DTI Study. Frontiers in Behavioral Neuroscience. 2018;12.

194. Li SS, Ma ZX, Tu SP, Zhou MK, Chen SH, Guo ZW, et al. Altered Resting-State Functional and White Matter Tract Connectivity in Stroke Patients With Dysphagia. Neurorehabilitation and Neural Repair. 2014;28(3):260-72.

195. Li YX, Wang Y, Zhang HY, Wu P, Huang WH. The Effect of Acupuncture on the Motor Function and White Matter Microstructure in Ischemic Stroke Patients. Evidence-Based Complementary and Alternative Medicine. 2015.

196. Li YX, Wu P, Liang FR, Huang WH. The Microstructural Status of the Corpus Callosum Is Associated with the Degree of Motor Function and Neurological Deficit in Stroke Patients. Plos One. 2015;10(4).

197. Liang Z, Zeng J, Zhang C, Liu S, Ling X, Xu A, et al. Longitudinal investigations on the anterograde and retrograde degeneration in the pyramidal tract following pontine infarction with diffusion tensor imaging. Cerebrovasc Dis. 2008;25(3):209-16.

198. Liegeois FJ, Butler J, Morgan AT, Clayden JD, Clark CA. Anatomy and lateralization of the human corticobulbar tracts: an fMRI-guided tractography study. Brain Struct Funct. 2016;221(6):3337-45.

199. Liegeois F, Tournier JD, Pigdon L, Connelly A, Morgan AT. Corticobulbar tract changes as predictors of dysarthria in childhood brain injury. Neurology. 2013;80(10):926-32.

200. Lin FX, Wu J, Zhao B, Tong XZ, Jin Z, Cao Y, et al. Preoperative Functional Findings and Surgical Outcomes in Patients with Motor Cortical Arteriovenous Malformation. World Neurosurgery. 2016;85:273-81.

201. Lin FX, Zhao B, Wu J, Wang LJ, Jin Z, Cao Y, et al. Risk factors for worsened muscle strength after the surgical treatment of arteriovenous malformations of the eloquent motor area. Journal of Neurosurgery. 2016;125(2):289-98.

202. Lin YC, Daducci A, Meskaldji DE, Thiran JP, Michel P, Meuli R, et al. Quantitative Analysis of Myelin and Axonal Remodeling in the Uninjured Motor Network After Stroke. Brain Connect. 2015;5(7):401-12.

203. Lin Y, Lin F, Kang D, Jiao Y, Cao Y, Wang S. Supratentorial cavernous malformations adjacent to the corticospinal tract: surgical outcomes and predictive value of diffusion tensor imaging findings. J Neurosurg. 2018;128(2):541-52.

204. Lindberg PG, Bensmail D, Bussel B, Maier MA, Feydy A. Wallerian Degeneration in Lateral Cervical Spinal Cord Detected with Diffusion Tensor Imaging in Four Chronic Stroke Patients. Journal of Neuroimaging. 2011;21(1):44-8.

205. Liu JC, Qin W, Zhang J, Zhang XJ, Yu CH. Enhanced Interhemispheric Functional Connectivity Compensates for Anatomical Connection Damages in Subcortical Stroke. Stroke. 2015;46(4):1045-51.

206. Liu X, Tian W, Li L, Kolar B, Qiu X, Chen F, et al. Hyperintensity on diffusion weighted image along ipsilateral cortical spinal tract after cerebral ischemic stroke: a diffusion tensor analysis. Eur J Radiol. 2012;81(2):292-7.

207. Liu YY, Chen L, Zeng JS, Li WM, Zeng SD, Ye B, et al. Proliferation of Bilateral Nerve Fibers Following Thalamic Infarction Contributes to Neurological Function Recovery: A Diffusion Tensor Imaging (DTI) Study. Medical Science Monitor. 2018;24:1464-72.

208. Lo R, Gitelman D, Levy R, Hulvershorn J, Parrish T. Identification of critical areas for motor function recovery in chronic stroke subjects using voxel-based lesion symptom mapping. Neuroimage. 2010;49(1):9-18.

209. Lotze M, Roschka S, Domin M, Platz T. Predicting Training Gain for a 3 Week Period of Arm Ability Training in the Subacute Stage After Stroke. Frontiers in Neurology. 2018;9.

210. Lu MK, Chen CM, Duann JR, Ziemann U, Chen JC, Chiou SM, et al. Investigation of Motor Cortical Plasticity and Corticospinal Tract Diffusion Tensor Imaging in Patients with Parkinsons Disease and Essential Tremor. PLoS One. 2016;11(9):e0162265.

211. Madhavan S, Krishnan C, Jayaraman A, Rymer WZ, Stinear JW. Corticospinal tract integrity correlates with knee extensor weakness in chronic stroke survivors. Clinical Neurophysiology. 2011;122(8):1588-94.

212. Madhavan S, Rogers LM, Stinear JW. A paradox: after stroke, the non-lesioned lower limb motor cortex may be maladaptive. European Journal of Neuroscience. 2010;32(6):1032-9.

213. Maesawa S, Fujii M, Nakahara N, Watanabe T, Wakabayashi T, Yoshida J. Intraoperative tractography and motor evoked potential (MEP) monitoring in surgery for gliomas around the corticospinal tract. World Neurosurg. 2010;74(1):153-61.

214. Magill ST, Han SJ, Li J, Berger MS. Resection of primary motor cortex tumors: feasibility and surgical outcomes. Journal of Neurosurgery. 2018;129(4):961-72.

215. Mah S, deVeber G, Wei XC, Liapounova N, Kirton A. Cerebellar Atrophy in Childhood Arterial Ischemic Stroke Acute Diffusion MRI Biomarkers. Stroke. 2013;44(9):2468-74.

216. Manganotti P, Storti SF, Formaggio E, Acler M, Zoccatelli G, Pizzini FB, et al. Effect of median-nerve electrical stimulation on BOLD activity in acute ischemic stroke patients. Clinical Neurophysiology. 2012;123(1):142-53.

217. Maximova MY, Popova TA, Konovalov RN. [Prsmall o, Cyrillicgnosis of motor function recovery in ischemic stroke using diffusion tensor MRI]. Zh Nevrol Psikhiatr Im S S Korsakova. 2016;116(8 Pt 2):57-64.

218. McClelland V, Mills K, Siddiqui A, Selway R, Lin JP. Central motor conduction studies and diagnostic magnetic resonance imaging in children with severe primary and secondary dystonia. Developmental Medicine and Child Neurology. 2011;53(8):757-63.

219. McDowell MM, Kellner CP, Barton SM, Mikell CB, Sussman ES, Heuts SG, et al. The role of advanced neuroimaging in intracerebral hemorrhage. Neurosurgical Focus. 2013;34(4).

220. Miao XL, Wang ZG, Yang WD, Zhao Y, Chen ZJ, Yu FH, et al. [Application of high-field intraoperative magnetic resonance imaging in stereotactic aspiration and drainage of hypertensive hematomas]. Zhonghua Yi Xue Za Zhi. 2012;92(35):2491-4.

221. Milot MH, Spencer SJ, Chan V, Allington JP, Klein J, Chou C, et al. Corticospinal Excitability as a Predictor of Functional Gains at the Affected Upper Limb Following Robotic Training in Chronic Stroke Survivors. Neurorehabilitation and Neural Repair. 2014;28(9):819-27.

222. Moon HI, Lee HJ, Yoon SY. Lesion location associated with balance recovery and gait velocity change after rehabilitation in stroke patients. Neuroradiology. 2017;59(6):609-18.

223. Morgan AT, Su M, Reilly S, Conti-Ramsden G, Connelly A, Liegeois FJ. A Brain Marker for Developmental Speech Disorders. Journal of Pediatrics. 2018;198:234-+.

224. Morita N, Wang SM, Kadakia P, Chawla S, Poptani H, Melhem ER. Diffusion Tensor Imaging of the Corticospinal Tract in Patients with Brain Neoplasms. Magnetic Resonance in Medical Sciences. 2011;10(4):239-43.

225. Mrachacz-Kersting N, Jiang N, Stevenson AJT, Niazi IK, Kostic V, Pavlovic A, et al. Efficient neuroplasticity induction in chronic stroke patients by an associative brain-computer interface. Journal of Neurophysiology. 2016;115(3):1410-21.

226. Nakashima A, Moriuchi T, Mitsunaga W, Yonezawa T, Kataoka H, Nakashima R, et al. Prediction of prognosis of upper-extremity function following stroke-related paralysis using brain imaging. J Phys Ther Sci. 2017;29(8):1438-43.

227. Nelles M, Urbach H, Sassen R, Schone-Bake J, Tschampa H, Traber F, et al. Functional hemispherectomy: postoperative motor state and correlation to preoperative DTI. Neuroradiology. 2015;57(11):1093-102.

228. Ng ASL, Sitoh YY, Zhao Y, Teng EWL, Tan EK, Tan LCS. Ipsilateral Stroke in a Patient With Horizontal Gaze Palsy With Progressive Scoliosis and a Subcortical Infarct. Stroke. 2011;42(1):E1-E3.

229. Ochoa JF, Ascencio JL, Suarez JC. [Application of advanced neuroimaging in motor rehabilitation]. Biomedica. 2014;34(3):330-9.

230. Panara V, Navarra R, Mattei PA, Piccirilli E, Bartoletti V, Uncini A, et al. Correlations between cervical spinal cord magnetic resonance diffusion tensor and diffusion kurtosis imaging metrics and motor performance in patients with chronic ischemic brain lesions of the corticospinal tract. Neuroradiology. 2019;61(2):175-82.

231. Park JW, Kim SH, Kim YW, Kim JY, Park SY, Son SM, et al. Motor control via spared peri-infarct corticospinal tract in patients with pontine infarct. Journal of Computer Assisted Tomography. 2008;32(1):159-62.

232. Peters DM, Fridriksson J, Stewart JC, Richardson JD, Rorden C, Bonilha L, et al. Cortical disconnection of the ipsilesional primary motor cortex is associated with gait speed and upper extremity motor impairment in chronic left hemispheric stroke. Human Brain Mapping. 2018;39(1):120-32.

233. Petoe MA, Byblow WD, de Vries EJM, Krishnamurthy V, Zhong CS, Barber PA, et al. A template-based procedure for determining white matter integrity in the internal capsule early after stroke. Neuroimage-Clinical. 2014;4:695-700.

234. Plow EB, Cunningham DA, Varnerin N, Machado A. Rethinking Stimulation of the Brain in Stroke Rehabilitation: Why Higher Motor Areas Might Be Better Alternatives for Patients with Greater Impairments. Neuroscientist. 2015;21(3):225-40.

235. Potter-Baker KA, Varnerin NM, Cunningham DA, Roelle SM, Sankarasubramanian V, Bonnett CE, et al. Influence of Corticospinal Tracts from Higher Order Motor Cortices on Recruitment Curve Properties in Stroke. Frontiers in Neuroscience. 2016;10.

236. Radlinska BA, Blunk Y, Leppert IR, Minuk J, Pike GB, Thiel A. Changes in callosal motor fiber integrity after subcortical stroke of the pyramidal tract. J Cereb Blood Flow Metab. 2012;32(8):1515-24.

237. Radlinska BA, Ghinani SA, Lyon P, Jolly D, Soucy JP, Minuk J, et al. Multimodal microglia imaging of fiber tracts in acute subcortical stroke. Ann Neurol. 2009;66(6):825-32.

238. Rados M, Nikic I, Kostovic I, Hof PR, Simic G. Functional reorganization oF the primary motor cortex in a patient with a large arteriovenous malFormation involving the precentral gyrus. Translational Neuroscience. 2013;4(2):269-72.

239. Ramu J, Herrera J, Grill R, Bockhorst T, Narayana P. Brain fiber tract plasticity in experimental spinal cord injury: Diffusion tensor imaging. Experimental Neurology. 2008;212(1):100-7.

240. Ressel V, Tuura RO, Scheer I, van Hedel HJA. Diffusion tensor imaging predicts motor outcome in children with acquired brain injury. Brain Imaging and Behavior. 2017;11(5):1373-84.

241. Rickards T, Sterling C, Taub E, Perkins-Hu C, Gauthier L, Graham M, et al. Diffusion Tensor Imaging Study of the Response to Constraint-Induced Movement Therapy of Children With Hemiparetic Cerebral Palsy and Adults With Chronic Stroke. Archives of Physical Medicine and Rehabilitation. 2014;95(3):506-14.

242. Rimmele DL, Frey BM, Cheng B, Schulz R, Krawinkel LA, Bonstrup M, et al. Association of Extrapyramidal Tracts' Integrity With Performance in Fine Motor Skills After Stroke. Stroke. 2018;49(12):2928-32.

243. Rocca MA, Turconi AC, Strazzer S, Absinta M, Valsasina P, Beretta E, et al. MRI Predicts Efficacy of Constraint-Induced Movement Therapy in Children With Brain Injury. Neurotherapeutics. 2013;10(3):511-9.

244. Rong DD, Zhang M, Ma QF, Lu J, Li KC. Corticospinal Tract Change during Motor Recovery in Patients with Medulla Infarct: A Diffusion Tensor Imaging Study. Biomed Research International. 2014.

245. Rosso C, Valabregue R, Attal Y, Vargas P, Gaudron M, Baronnet F, et al. Contribution of Corticospinal Tract and Functional Connectivity in Hand Motor Impairment after Stroke. Plos One. 2013;8(9).

246. Roze E, Benders MJ, Kersbergen KJ, van der Aa NE, Groenendaal F, van Haastert IC, et al. Neonatal DTI early after birth predicts motor outcome in preterm infants with periventricular hemorrhagic infarction. Pediatric Research. 2015;78(3):298-303.

247. Roze E, Harris PA, Ball G, Elorza LZ, Braga RM, Allsop JM, et al. Tractography of the corticospinal tracts in infants with focal perinatal injury: comparison with normal controls and to motor development. Neuroradiology. 2012;54(5):507-16.

248. Ruber T, Lindenberg R, Schlaug G. Differential Adaptation of Descending Motor Tracts in Musicians. Cerebral Cortex. 2015;25(6):1490-8.

249. Saada F, Antonios N. Existence of ipsilateral hemiparesis in ischemic and hemorrhagic stroke: two case reports and review of the literature. Eur Neurol. 2014;71(1-2):25-31.

250. Schaechter JD, Perdue KL, Wang RP. Structural damage to the corticospinal tract correlates with bilateral sensorimotor cortex reorganization in. stroke patients. Neuroimage. 2008;39(3):1370-82.

251. Schertz M, Shiran SI, Myers V, Weinstein M, Fattal-Valevski A, Artzi M, et al. Imaging Predictors of Improvement From a Motor Learning-Based Intervention for Children With Unilateral Cerebral Palsy. Neurorehabilitation and Neural Repair. 2016;30(7):647-60.

252. Schulz R, Braass H, Liuzzi G, Hoerniss V, Lechner P, Gerloff C, et al. White matter integrity of premotor-motor connections is associated with motor output in chronic stroke patients. Neuroimage-Clinical. 2015;7:82-6.

253. Schulz R, Park E, Lee J, Chang WH, Lee A, Kim YH, et al. Interactions Between the Corticospinal Tract and Premotor-Motor Pathways for Residual Motor Output After Stroke. Stroke. 2017;48(10):2805-+.

254. Schulz R, Park E, Lee J, Chang WH, Lee A, Kim YH, et al. Synergistic but independent: The role of corticospinal and alternate motor fibers for residual motor output after stroke. Neuroimage-Clinical. 2017;15:118-24.

255. Seo JP, Chang PH, Jang SH. Anatomical location of the corticospinal tract according to somatotopies in the centrum semiovale. Neuroscience Letters. 2012;523(2):111-4.

256. Seo JP, Do KH, Jung GS, Seo SW, Kim K, Son SM, et al. The difference of gait pattern according to the state of the corticospinal tract in chronic hemiparetic stroke patients. Neurorehabilitation. 2014;34(2):259-66.

257. Seo JP, Jang SH. Different Characteristics of the Corticospinal Tract According to the Cerebral Origin: DTI Study. American Journal of Neuroradiology. 2013;34(7):1359-63.

258. Seo JP, Jang SH. Recovery of the corticospinal tracts injured by subfalcine herniation: a diffusion tensor tractography study. Neural Regeneration Research. 2014;9(12):1231-3.

259. Seo JP, Lee MY, Kwon YH, Jang SH. Delayed gait recovery in a stroke patient(star)star(.). Neural Regeneration Research. 2013;8(16):1514-8.

260. Seo YS, Jang SH. Recovery of a degenerated corticospinal tract after injury in a patient with intracerebral hemorrhage: confirmed by diffusion tensor tractography imaging. Neural Regeneration Research. 2015;10(5):829-31.

261. Shin HE, Suh HC, Kang SH, Seo KM, Kim DK, Shin HW. Diagnostic Challenge of Diffusion Tensor Imaging in a Patient With Hemiplegia After Traumatic Brain Injury. Annals of Rehabilitation Medicine-Arm. 2017;41(1):153-7.

262. Shin SS, Verstynen T, Pathak S, Jarbo K, Hricik AJ, Maserati M, et al. High-definition fiber tracking for assessment of neurological deficit in a case of traumatic brain injury: finding, visualizing, and interpreting small sites of damage. J Neurosurg. 2012;116(5):1062-9.

263. Skrap M, Vescovi MC, Uletto GAP, Maieron M, Tomasino B, Bagatto D, et al. Supratentorial Cavernous Malformations Involving the Corticospinal Tract and Sensory Motor Cortex: Treatment Strategies, Surgical Considerations, and Outcomes. Operative Neurosurgery. 2018;15(5):483-97.

264. Spampinato MV, Chan C, Jensen JH, Helpern JA, Bonilha L, Kautz SA, et al. Diffusional Kurtosis Imaging and Motor Outcome in Acute Ischemic Stroke. American Journal of Neuroradiology. 2017;38(7):1328-34.

265. Stefanou MI, Lumsden DE, Ashmore J, Ashkan K, Lin JP, Charles-Edwards G. Tensor and non-tensor tractography for the assessment of the corticospinal tract of children with motor disorders: a comparative study. Neuroradiology. 2016;58(10):1005-16.

266. Steinbach R, Loewe K, Kaufmann J, Machts J, Kollewe K, Petri S, et al. Structural hallmarks of amyotrophic lateral sclerosis progression revealed by probabilistic fiber tractography. Journal of Neurology. 2015;262(10):2257-70.

267. Sterr A, Dean PJA, Szameitat AJ, Conforto AB, Shen S. Corticospinal Tract Integrity and Lesion Volume Play Different Roles in Chronic Hemiparesis and Its Improvement Through Motor Practice. Neurorehabilitation and Neural Repair. 2014;28(4):335-43.

268. Takebayashi T, Marumoto K, Takahashi K, Domen K. Differences in neural pathways are related to the short- or long-term benefits of constraint-induced movement therapy in patients with chronic stroke and hemiparesis: a pilot cohort study. Topics in Stroke Rehabilitation. 2018;25(3):203-8.

269. Tang PF, Ko YH, Luo ZA, Yeh FC, Chen SHA, Tseng WYI. Tract-Specific and Region of Interest Analysis of Corticospinal Tract Integrity in Subcortical Ischemic Stroke: Reliability and Correlation with Motor Function of Affected Lower Extremity. American Journal of Neuroradiology. 2010;31(6):1023-30.

270. Taniguchi A, Ii Y, Kawana Y, Asahi M, Naito Y, Shibata M, et al. [Case of ipsilateral monoparesis by lacunar infarction: a consideration on the pathological mechanism]. Brain Nerve. 2011;63(2):177-80.

271. Terceno M, Puig J, Blasco G, Daunis-i-Estadella J, Hernandez-Perez M, Serena J, et al. Acute motor deficit and early corticospinal tract damage on diffusion tensor imaging predict motor outcome after intracerebral hemorrhage. International Journal of Stroke. 2015;10:58-9.

272. Thiel A, Vahdat S. Structural and Resting-State Brain Connectivity of Motor Networks After Stroke. Stroke. 2015;46(1):296-301.

273. Tovar-Moll F, Evangelou IE, Chiu AW, Auh S, Chen C, Ehrmantraut M, et al. Diffuse and focal corticospinal tract disease and its impact on patient disability in multiple sclerosis. J Neuroimaging. 2015;25(2):200-6.

274. Ueda R, Yamada N, Kakuda W, Abo M, Senoo A. White matter structure and clinical characteristics of stroke patients: A diffusion tensor MRI study. Brain Research. 2016;1635:61-70.

275. van der Aa NE, Leemans A, Northington FJ, van Straaten HL, van Haastert IC, Groenendaal F, et al. Does Diffusion Tensor Imaging-Based Tractography at 3 Months of Age Contribute to the Prediction of Motor Outcome After Perinatal Arterial Ischemic Stroke? Stroke. 2011;42(12):3410-4.

276. Vargas P, Gaudron M, Valabregue R, Bertasi E, Humbert F, Lehericy S, et al. Assessment of corticospinal tract (CST) damage in acute stroke patients: Comparison of tract-specific analysis versus segmentation of a CST template. Journal of Magnetic Resonance Imaging. 2013;37(4):836-45.

277. Wagenaar N, van der Aa NE, Groenendaal F, Verhage CH, Benders M, de Vries LS. MR imaging for accurate prediction of outcome after perinatal arterial ischemic stroke: Sooner not necessarily better. European Journal of Paediatric Neurology. 2017;21(4):666-70.

278. Wang AC, Ibrahim GM, Poliakov AV, Wang PI, Fallah A, Mathern GW, et al. Corticospinal tract atrophy and motor fMRI predict motor preservation after functional cerebral hemispherectomy. Journal of Neurosurgery-Pediatrics. 2018;21(1):81-9.

279. Wang LE, Tittgemeyer M, Imperati D, Diekhoff S, Ameli M, Fink GR, et al. Degeneration of corpus callosum and recovery of motor function after stroke: A multimodal magnetic resonance imaging study. Human Brain Mapping. 2012;33(12):2941-56.

280. Wang RR, Li C, Zhang S, Zhou LJ, He L, Li HD. Diffusion tensor imaging change in crus cerebri in striatocapsular infarction and correlation with upper extremity motor dysfunction. Radiol Med. 2015;120(11):1064-70.

281. Wilkins EG, Kamel H, Johnson EC, Shalev SM, Josephson SA. Ischemic stroke of the pyramidal decussation causing quadriplegia and anarthria. J Stroke Cerebrovasc Dis. 2012;21(7):620.e1-2.

282. Wu GF, Wang LK, Liu J, Mao YH, Qin GN. Minimally Invasive Procedures Reduced the Damages to Motor Function in Patients with Thalamic Hematoma: Observed by Motor Evoked Potential and Diffusion Tensor Imaging. Journal of Stroke & Cerebrovascular Diseases. 2013;22(3):232-40.

283. Wu XN, Zhang T, Wang J, Liu XY, Li ZS, Xiang W, et al. Magnetic resonance diffusion tensor imaging following major ozonated autohemotherapy for treatment of acute cerebral infarction. Neural Regeneration Research. 2016;11(7):1115-21.

284. Xi SD, Zhu YL, Chen C, Liu HQ, Wang WW, Li F. The plasticity of the corticospinal tract in children with obstetric brachial plexus palsy after Botulinum Toxin A treatment. Journal of the Neurological Sciences. 2018;394:19-25.

285. Xu SW, Chen ZQ, Chen JH, Ye YQ, Lin YN, Ni P, et al. Correlation between muscular strength and basal nuclei ischemic/hemorrhagic stroke-induced corticospinal tract injury, as detected by diffusion tensor imaging and tractography. Neural Regeneration Research. 2010;5(13):1010-4.

286. Yamada N, Ueda R, Kakuda W, Momosaki R, Kondo T, Hada T, et al. Diffusion Tensor Imaging Evaluation of Neural Network Development in Patients Undergoing Therapeutic Repetitive Transcranial Magnetic Stimulation following Stroke. Neural Plasticity. 2018.

287. Yamamoto Y, Nagakane Y, Tomii Y, Toda S, Akiguchi I. The relationship between progressive motor deficits and lesion location in patients with single infarction in the lenticulostriate artery territory. J Neurol. 2017;264(7):1381-7.

288. Yang DS, Hong JH, Byun WM, Kwak SY, Ahn SH, Lee H, et al. Identification of the medial lemniscus in the human brain: Combined study of functional MRI and diffusion tensor tractography. Neuroscience Letters. 2009;459(1):19-24.

289. Yeo SS, Ahn SH, Choi BY, Chang CH, Lee J, Jang SH. Contribution of the Pedunculopontine Nucleus on Walking in Stroke Patients. European Neurology. 2011;65(6):332-7.

290. Yeo SS, Choi BY, Chang CH, Kim SH, Jung YJ, Jang SH. Evidence of Corticospinal Tract Injury at Midbrain in Patients With Subarachnoid Hemorrhage. Stroke. 2012;43(8):2239-41.

291. Yeo SS, Jang SH. Changes in red nucleus after pyramidal tract injury in patients with cerebral infarct. Neurorehabilitation. 2010;27(4):373-7.

292. Yeo SS, Jang SH. Motor recovery via aberrant pyramidal tract in a patient with a cerebral peduncle infarct. Neural Regeneration Research. 2011;6(13):1023-6.

293. Yeo SS, Jang SH. Reorganization of corticospinal tract in a patient with posterior limb infarct of the internal capsule. Neural Regeneration Research. 2011;6(4):313-6.

294. Yeo SS, Jang SH. A change in injured corticospinal tract originating from the premotor cortex to the primary motor cortex in a patient with intracerebral hemorrhage. Neural Regeneration Research. 2012;7(12):939-42.

295. Yeo SS, Jang SH. Differences between the somatotopic corticospinal tract for the fingers and toes in the human brain. Neurorehabilitation. 2012;31(4):395-9.

296. Yeo SS, Jang SH. Ipsilateral motor pathway without contralateral motor pathway in a stroke patient. Neurorehabilitation. 2012;30(4):303-6.

297. Yeo SS, Jang SH. Corticospinal tract recovery in a patient with traumatic transtentorial herniation. Neural Regeneration Research. 2013;8(5):469-73.

298. Yeo SS, Jang SH. Motor recovery via aberrant pyramidal tract in a patient with traumatic brain injury A diffusion tensor tractography study. Neural Regeneration Research. 2013;8(1):90-4.

299. Yeo SS, Jang SH. Recovery of an injured corticospinal tract and an injured corticoreticular pathway in a patient with intracerebral hemorrhage. Neurorehabilitation. 2013;32(2):305-9.

300. Yeo SS, Jang SH, Son SM. The different maturation of the corticospinal tract and corticoreticular pathway in normal brain development: diffusion tensor imaging study. Frontiers in Human Neuroscience. 2014;8.

301. Yeo SS, Kim SH, Jang SH. Proximal weakness due to injury of the corticoreticular pathway in a patient with traumatic brain injury. Neurorehabilitation. 2013;32(3):665-9.

302. Yin D, Yan X, Fan M, Hu Y, Men W, Sun L, et al. Secondary degeneration detected by combining voxel-based morphometry and tract-based spatial statistics in subcortical strokes with different outcomes in hand function. AJNR Am J Neuroradiol. 2013;34(7):1341-7.

303. Yokoyama K, Matsuki M, Shimano H, Sumioka S, Ikenaga T, Hanabusa K, et al. Diffusion tensor imaging in chronic subdural hematoma: correlation between clinical signs and fractional anisotropy in the pyramidal tract. AJNR Am J Neuroradiol. 2008;29(6):1159-63.

304. Yoo JS, Choi BY, Chang CH, Jung YJ, Kim SH, Jang SH. Characteristics of injury of the corticospinal tract and corticoreticular pathway in hemiparetic patients with putaminal hemorrhage. Bmc Neurology. 2014;14.

305. Yoo WK, Kim DS, Kwon YH, Jang SH. Kernohan's notch phenomenon demonstrated by diffusion tensor imaging and transcranial magnetic stimulation. J Neurol Neurosurg Psychiatry. 2008;79(11):1295-7.

306. Young JA, Tolentino M. Neuroplasticity and Its Applications for Rehabilitation. American Journal of Therapeutics. 2011;18(1):70-80.

307. Young RJ, Lee V, Peck KK, Sierra T, Zhang ZG, Jacks LM, et al. Diffusion Tensor Imaging and Tractography of the Corticospinal Tract in the Presence of Enlarged Virchow-Robin Spaces. Journal of Neuroimaging. 2014;24(1):79-82.

308. Yu S, Carlson HL, Mineyko A, Brooks BL, Kuczynski A, Hodge J, et al. Bihemispheric alterations in myelination in children following unilateral perinatal stroke. Neuroimage-Clinical. 2018;20:7-15.

309. Yu XF, Song RR, Jiaerken Y, Yuan LX, Huang PY, Lou M, et al. White matter injury induced by diabetes in acute stroke is clinically relevant: A preliminary study. Diabetes & Vascular Disease Research. 2017;14(1):40-6.

310. Zhang F, Lu GM, Zee CS. Comparative study of the sensitivity of ADC value and T-2 relaxation time for early detection of Wallerian degeneration. European Journal of Radiology. 2011;79(1):118-23.

311. Zhang LD, Butler AJ, Sun CK, Sahgal V, Wittenberg GF, Yue GH. Fractal dimension assessment of brain white matter structural complexity post stroke in relation to upper-extremity motor function. Brain Research. 2008;1228:229-40.

312. Zhang RZ, Tao CY, Chen W, Wang CH, Hu Y, Song L, et al. Dynamic Diffusion Tensor Imaging Reveals Structural Changes in the Bilateral Pyramidal Tracts after Brain Stem Hemorrhage in Rats. Front Neuroanat. 2016;10:33.

313. Zhang Y, Wan S, Zhang X. Geniculocalcarine Tract Disintegration after Ischemic Stroke: A Diffusion Tensor Imaging Study. American Journal of Neuroradiology. 2013;34(10):1890-4.

314. Zhao N, Zhang JN, Qiu MG, Wang CR, Xiang Y, Wang H, et al. Scalp acupuncture plus low-frequency rTMS promotes repair of brain white matter tracts in stroke patients: A DTI study. Journal of Integrative Neuroscience. 2018;17(1):61-9.

315. Zheng XY, Chen DF, Yan TB, Jin DM, Zhuang ZQ, Tan ZM, et al. A Randomized Clinical Trial of a Functional Electrical Stimulation Mimic to Gait Promotes Motor Recovery and Brain Remodeling in Acute Stroke. Behavioural Neurology. 2018.

316. Zheng X, Schlaug G. Structural white matter changes in descending motor tracts correlate with improvements in motor impairment after undergoing a treatment course of tDCS and physical therapy. Frontiers in Human Neuroscience. 2015;9.

317. Zhou Y, Zhang RT, Zhang S, Yan SQ, Wang Z, Campbell BCV, et al. Impact of perfusion lesion in corticospinal tract on response to reperfusion. European Radiology. 2017;27(12):5280-9.

**Manuscripts excluded by abstract**

1. Axer H, Axer M, Grassel D, Witte OW. Imaging of Wallerian degeneration in the brain. Current Medical Imaging Reviews. 2008;4(2):71-6.

2. Borich MR, Mang C, Boyd LA. Both projection and commissural pathways are disrupted in individuals with chronic stroke: investigating microstructural white matter correlates of motor recovery. Bmc Neuroscience. 2012;13.

3. Borich MR, Wadden KP, Boyd LA. Establishing the reproducibility of two approaches to quantify white matter tract integrity in stroke. Neuroimage. 2012;59(3):2393-400.

4. Buetefisch CM, Revill KP, Haut MW, Kowalski GM, Wischnewski M, Pifer M, et al. Abnormally reduced primary motor cortex output is related to impaired hand function in chronic stroke. Journal of Neurophysiology. 2018;120(4):1680-94.

5. Cassidy JM, Tran G, Quinlan EB, Cramer SC. Neuroimaging Identifies Patients Most Likely to Respond to a Restorative Stroke Therapy. Stroke. 2018;49(2):433-8.

6. Feldman SJ, Boyd LA, Neva JL, Peters S, Hayward KS. Extraction of corticospinal tract microstructural properties in chronic stroke. Journal of Neuroscience Methods. 2018;301:34-42.

7. Guggisberg AG, Nicolo P, Cohen LG, Schnider A, Buch ER. Longitudinal Structural and Functional Differences Between Proportional and Poor Motor Recovery After Stroke. Neurorehabilitation and Neural Repair. 2017;31(12):1029-41.

8. Hong JH, Jang SH. Motor recovery by anterior choroidal artery territory in a patient with middle cerebral artery infarct. Neural Regeneration Research. 2010;5(17):1357-60.

9. Hong JH, Jang SH. Aberrant pyramidal tract in a patient with corona radiata infarct A diffusion tensor tractography study. Neural Regeneration Research. 2011;6(13):1027-30.

10. Jang SH, Chang MC. Motor outcomes of patients with a complete middle cerebral artery territory infarct. Neural Regeneration Research. 2013;8(20):1892-7.

11. Jang SH, Kim K, Kim SH, Son SM, Jang WH, Kwon HG. The relation between motor function of stroke patients and diffusion tensor imaging findings for the corticospinal tract. Neuroscience Letters. 2014;572:1-6.

12. Jang SH, Park JW, Choi BY, Kim SH, Chang CH, Jung YJ, et al. Difference of recovery course of motor weakness according to state of corticospinal tract in putaminal hemorrhage. Neuroscience Letters. 2017;653:163-7.

13. Jung YJ, Jang SH. The Fate of Injured Corticospinal Tracts in Patients with Intracerebral Hemorrhage: Diffusion Tensor Imaging Study. American Journal of Neuroradiology. 2012;33(9):1775-8.

14. Kim AR, Kim DH, Park SY, Kyeong S, Kim YW, Lee SK, et al. Can the integrity of the corticospinal tract predict the long-term motor outcome in poststroke hemiplegic patients? Neuroreport. 2018;29(6):453-8.

15. Kim B, Fisher BE, Schweighofer N, Leahy RM, Haldar JP, Choi S, et al. A comparison of seven different DTI-derived estimates of corticospinal tract structural characteristics in chronic stroke survivors. Journal of Neuroscience Methods. 2018;304:66-75.

16. Kim H, Lee H, Jung KI, Ohn SH, Yoo WK. Changes in Diffusion Metrics of the Red Nucleus in Chronic Stroke Patients With Severe Corticospinal Tract Injury: A Preliminary Study. Annals of Rehabilitation Medicine-Arm. 2018;42(3):396-405.

17. Kim SK, Song P, Hong JM, Pak CY, Chung CS, Lee KH, et al. Prediction of progressive motor deficits in patients with deep subcortical infarction. Cerebrovasc Dis. 2008;25(4):297-303.

18. Koyama T, Marumoto K, Miyake H, Ohmura T, Domen K. Relationship between diffusion-tensor fractional anisotropy and long-term outcome in patients with hemiparesis after intracerebral hemorrhage. Neurorehabilitation. 2013;32(1):87-94.

19. Lindow J, Domin M, Grothe M, Horn U, Eickhoff SB, Lotze M. Connectivity-Based Predictions of Hand Motor Outcome for Patients at the Subacute Stage After Stroke. Frontiers in Human Neuroscience. 2016;10.

20. Liu G, Tan SQ, Dang C, Peng KQ, Xie CM, Xing SH, et al. Motor Recovery Prediction With Clinical Assessment and Local Diffusion Homogeneity After Acute Subcortical Infarction. Stroke. 2017;48(8):2121-+.

21. Liu HM, Lai C, Zhang SZ. [White matter tractography by diffusion tensor imaging in prognosis of acute lacunar infarctions]. Zhejiang Da Xue Xue Bao Yi Xue Ban. 2009;38(2):186-93.

22. Lugokenski R, Welter LD, Pasqualotti A, Biancini R, Lucatelli V. MR-Tractography in the Evaluation of the Rehabilitation Process in Stroke Patients. In: Rocha A, Dias GP, Martins A, Reis LP, Cota MP, editors. 2015 10th Iberian Conference on Information Systems and Technologies. Iberian Conference on Information Systems and Technologies2015.

23. Maraka S, Jiang Q, Jafari-Khouzani K, Hamidian N, Li L, Malik S, et al. Quantitative Assessment of Corticospinal Tract Injury on Diffusion Tensor Imaging Correlates with Motor Outcome after Ischemic Stroke. Neurology. 2013;80.

24. Moulton E, Valabregue R, Diaz B, Kemlin C, Leder S, Lehericy S, et al. Comparison of spatial normalization strategies of diffusion MRI data for studying motor outcome in subacute-chronic and acute stroke. Neuroimage. 2018;183:186-99.

25. Oh S, Bang OY, Chung CS, Lee KH, Chang WH, Kim GM. Topographic Location of Acute Pontine Infarction Is Associated With the Development of Progressive Motor Deficits. Stroke. 2012;43(3):708-+.

26. Ohara T, Yamamoto Y, Tamura A, Ishii R, Murai T. The infarct location predicts progressive motor deficits in patients with acute lacunar infarction in the lenticulostriate artery territory. Journal of the Neurological Sciences. 2010;293(1-2):87-91.

27. Qiu MG, Darling WG, Morecraft RJ, Ni CC, Rajendra J, Butler AJ. White Matter Integrity Is a Stronger Predictor of Motor Function Than BOLD Response in Patients With Stroke. Neurorehabilitation and Neural Repair. 2011;25(3):275-84.

28. Seitz RJ, Donnan GA. Role of Neuroimaging in Promoting Long-Term Recovery From Ischemic Stroke. Journal of Magnetic Resonance Imaging. 2010;32(4):756-72.

29. Song F, Zhang F, Yin DZ, Hu YS, Fan MX, Ni HH, et al. Diffusion Tensor Imaging for Predicting Hand Motor Outcome in Chronic Stroke Patients. Journal of International Medical Research. 2012;40(1):126-33.

30. Sterr A, Shen S, Szameitat AJ, Herron KA. The Role of Corticospinal Tract Damage in Chronic Motor Recovery and Neurorehabilitation:A Pilot Study. Neurorehabilitation and Neural Repair. 2010;24(5):413-9.

31. Venkatasubramanian C, Kleinman JT, Fischbein NJ, Olivot JM, Gean AD, Eyngorn I, et al. Natural History and Prognostic Value of Corticospinal Tract Wallerian Degeneration in Intracerebral Hemorrhage. Journal of the American Heart Association. 2013;2(4).

32. Wei WJ, Bai LJ, Wang J, Dai RW, Tong RKY, Zhang YM, et al. A Longitudinal Study of Hand Motor Recovery after Sub-Acute Stroke: A Study Combined fMRI with Diffusion Tensor Imaging. Plos One. 2013;8(5).

33. Wen HM, Alshikho MJ, Wang Y, Luo X, Zafonte R, Herbert MR, et al. Correlation of Fractional Anisotropy With Motor Recovery in Patients With Stroke After Postacute Rehabilitation. Archives of Physical Medicine and Rehabilitation. 2016;97(9):1487-95.

34. Yang DS, Kim DS, Kim YH, Jang SH. Demonstration of recovery of a severely damaged corticospinal tract: A diffusion tensor tractography and transcranial magnetic stimulation follow-up study. Journal of Computer Assisted Tomography. 2008;32(3):418-20.

35. Yeo SS, Choi BY, Chang CH, Jang SH. Transpontine Connection Fibers between Corticospinal Tracts in Hemiparetic Patients with Intracerebral Hemorrhage. European Neurology. 2010;63(3):154-8.

36. Zeng J, Zheng P, Xu J, Tong W, Guo Y, Yang W, et al. Prediction of motor function by diffusion tensor tractography in patients with basal ganglion haemorrhage. Arch Med Sci. 2011;7(2):310-4.

37. Zhong CG, Bai LJ, Cui FY, Dai RW, Xue T, Wang H, et al. Tractography of white matter based on diffusion tensor imaging in ischaemic stroke involving the corticospinal tract: a preliminary study. In: Molthen RC, Weaver JB, editors. Medical Imaging 2012: Biomedical Applications in Molecular, Structural, and Functional Imaging. Proceedings of SPIE. 83172012.

**Supplementary Table – reasons for exclusion of studies from the review, after reading the manuscripts**

| **First author_Year** | **Reference** |
| --- | --- |
| **Not longitudinal studies** | |
| Archer_2017 | Human Brain Mapping. 2017; 38:4546–456 |
| Auriat_2015 | NeuroImage: Clinical. 2015; 7:771–781 |
| Chaudhary_2015 | Exp Neurol. 2015; 272:88–96 |
| Chen_2013 | Front Sys Neurol. 2013; 4:178 |
| Chun_2016 | Ann Rehabil Med. 2016; 40(1):126-134 |
| Filatova_2018 | Front Neurosc. 2018; 12:247 |
| Hayward_2017 | NeuroImage: Clinical. 2017; 13:310–319 |
| Hirai_2016 | J of Neurosc Met. 2016; 257:204–213 |
| Horn_2016 | Neural Plasticity. 2016; 1-12 |
| Jin_2017 | World J Emerg Med. 2017; 8(2):99–105 |
| Kim_2016 | Neurorehab and Neural Repair. 2017; 31(1):3-24 |
| Koyama_2018 | J Stroke Cerebrovasc Dis. 2018; 27(4):878-885 |
| Kumar_2015 | Journal of Stroke. 2016; 18(1):50-59 |
| Lindenberg_2010 | Neurology. 2010; 74:280–287 |
| Liu_2010 | Acta Neurol Scand. 2010; 121:315–319 |
| Liu_2012 | J Neuroimaging. 2012 ;22:255-260 |
| Nelles_2008 | Am J Neuroradiol. 2008; 29:488 –93 |
| Pannek_2009 | J Magn Reson Imaging. 2009; 29:529–536 |
| Park_2013 | NeuroImage: Clinical. 2013; 2:521–533 |
| Puig_2017 | Neuroradiology. 2017; 59:343–351 |
| Ruber_2012 | Neurology. 2012; 79:515–522 |

| **First author_Year** | **Reference** |
| --- | --- |
| **Lack of correlation between at least one DTI metric in the corticospinal tract collected up to 90 days post-stroke and motor outcomes assessed later than 4 weeks post-stroke** | |
| Bigourdan_2016 | Stroke. 2016; 47:1053-1059 |
| Byblow_2015 | Ann Neurol. 2015; 78:848–859 |
| Jang_2008 | Ann Neurol. 2008; 64:460–465 |
| Jang_2010 | J of the Neurol Sci. 2010; 290:107–111 |
| Kwon_2011 | J Rehabil Med. 2011; 43:430–434 |
| Kwon_2012 | Neuroradiology (2012) 54:691–697 |
| Liang_2009 | Neurorehab Neural Repair. 2009; 23(7):692-698 |
| Liu_2015 | Rest Neurol and Neurosc. 2015; 33(3):309-19 |
| Liu_2018 | Rest Neurol and Neurosc. 2018; 36:173–182 |
| Maraka_2014 | Annals of Clinical and Translational Neurology 2014; 1(11): 891–899 |
| Riley_2011 | Stroke. 2011; 42(2): 421–426 |
| Rosso_2011 | Journal of Neuroradiology. 2011; 38:105—112 |
| Snow_2016 | J Neurosc Meth. 2016; 257:109–120 |
| Sue_2017 | Neuroscience Letters. 2017; 658:155-160 |
| Takenobu_2014 | NeuroImage: Clinical. 2014; 4:201–208 |
| Zhu_2010 | Stroke. 2010; 41(5): 910–915 |
| **Lack of evaluation of motor outcomes at least 4 weeks after stroke** | |
| Fan_2015 | Rest Neurol Neurosc. 2015; 33: 835–844 |
| Grassel_2010 | Cerebrovasc Dis. 2010; 30:380–388 |
| Maeshima_2013 | Neurol Sci. 2013; 34:1765–1770 |
| Schulz_2012 | Stroke. 2012; 43(8):2248–2251 |
| Yang_2014 | J Neurol Sci. 2015; 359:219–225 |
|  | |
| **Sample size < 10** | |
| Lotze_2012 | Neurorehab and Neural Repair. 2012; 26(6):594–603 |
| Mang_2015 | Clinical Neurophysiology.2015; 126:1959–1971 |
| Marumoto_2013 | Restorative Neurology and Neuroscience. 2013; 31:387–396 |
| Schaechter_2009 | Hum Brain Mapp. 2009; 30(11):3461–3474 |
| Stewart_2017a | Neurorehab and Neural Repair. 2017; 31(7):657–665 |
| Stewart_2017b | NeuroImage: Clinical. 2017; 14:641–647 |
| Yang_2015 | PLoS ONE. 2015; 10(5):e0125038 |
| Young_2016 | Front Hum Neurosc. 2016; 10:457 |
| Yu_2009 | NeuroImage. 2009; 47:451–458 |
|  | |
| **DTI data collected in the chronic phase after stroke** | |
| Lindenberg_2012 | Hum Brain Mapp. 2012; 33(5): 1040–1051 |
