# Supplementary figures and images for: Diffusion Tensor Imaging Biomarkers to Predict Motor Outcomes in Stroke: A Narrative Review
Source: Front Neurol. 2019 May 8;10:445. doi: 10.3389/fneur.2019.00445 (PMC6530391; doi:10.3389/fneur.2019.00445)

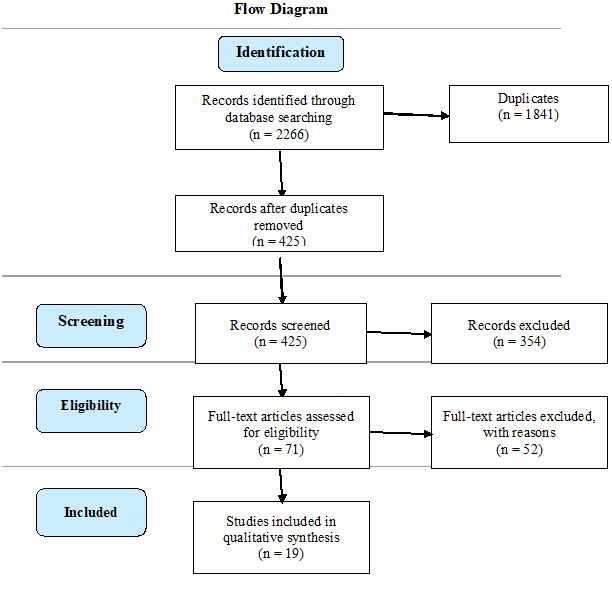

Supplement: Supplementary file 2 [file Image_1.TIF]
